# High-frequency diatom dynamics seen in an ice- and snow-covered temperate lake using an imaging-in-flow cytometer

**DOI:** 10.1007/s10750-025-05802-8

**Published:** 2025-02-06

**Authors:** Tara Tapics, Irene Gregory-Eaves, Yannick Huot

**Affiliations:** 1 Département de géomatique appliquée, Université de Sherbrooke, 2500, boul. de l’Université, Sherbrooke, QC CA J1K 2R1 Canada; 2 Department of Biology, Faculty of Science, McGill University, 1205 Dr Penfield Ave, Montreal, QC CA H3A 1B1 Canada

**Keywords:** *Asterionella*, cf. *Synedra*, Imaging FlowCytobot (IFCB), *Urosolenia*, Under-ice, Winter ecology, Winter limnology

## Abstract

The study of winter in temperate, ice-covered lakes has largely been neglected, creating a major gap in our understanding of annual phytoplankton cycles. We assessed patterns in biovolumes of *Asterionella,* cf. *Synedra, Urosolenia,* and total phytoplankton at three depths during the winter of 2014/2015 as estimated using images from an Imaging FlowCytobot (IFCB) moored in Lac (Lake) Montjoie, Quebec (Canada). Even though the use of the IFCB under-ice presented challenges, these were outstripped by its advantages. The IFCB provided unprecedented high temporal and spatial resolution phytoplankton count and biovolume data that allowed patterns of abundance to be observed in detail. Interestingly, the IFCB captured an abundance of *Urosolenia*, which may be dissolved in standard Lugol’s-preserved grab samples. We found *Asterionella* grew until mid-December and then slowly decreased towards the spring, whereas cf. *Synedra* diatoms decreased from early December and then rebounded towards late December as light increased before falling again towards the spring. *Urosolenia* peaked in late December as cf. *Synedra* diatoms rebounded and then began to decline towards the spring. Total phytoplankton declined towards late December, increased in the beginning of January and then declined towards the spring. The studied diatoms maintained considerable under-ice seed populations and grew when light was sufficient.

**Supplementary Information:**

The online version contains supplementary material available at 10.1007/s10750-025-05802-8.

## Introduction

Winter phytoplankton communities in lakes with at least seasonal snow and ice cover can be difficult—and sometimes dangerous—to sample (Block et al., 2018). Nonetheless, they have been attracting increasing attention from researchers (Powers & Hampton, 2016). This shift away from the long-standing focus on the more easily accessible, generally higher productivity ice-free seasons (Cotner et al., 2022) comes alongside the recognition that annual phytoplankton cycles cannot be understood without reference to the winter season (Twiss et al., 2012; Hampton et al., 2015).

We therefore need to overcome the difficulties inherent to winter sampling in order to better characterise seasonal phytoplankton dynamics. At present, the lack of baseline information about the winter season means we are not well placed to make predictions about how changes in climate will affect the future states of lake phytoplankton communities. One approach to improving winter data collection is to deploy automated under-ice phytoplankton sampling platforms such as imaging-in-flow cytometers. Sample collection frequency for automated instruments like imaging-in-flow cytometers (Dashkova et al., 2017) outstrips what can be gathered manually by thousands of times. For example, in situ sensors can image the contents of more than 50 discrete water samples per day and run continuously over months, compared to a typical one discrete sample per week for manual sampling programs, or tens of samples over a single day for an intensive effort. Using automated systems for sampling also removes the difficulties and dangers inherent in winter field work on lakes.

One of the more evident climate-based shifts affecting lakes is a change in ice- and snow-cover dynamics (Benson et al., 2012; Sharma et al., 2016, 2020). Ice-on and ice-off times have already been altered (Huang et al., 2022; Woolway et al., 2022), as have patterns of snow and ice thickness on lakes (Sharma et al., 2016). The under-ice light environment is affected in turn (Cavaliere et al., 2021). Snow and ice (if lacking clarity) can be very effective barriers to the penetration of already low levels of light present during short winter days (Wright, 1964; Kirillin et al., 2012; Bramburger et al., 2022; Weyhenmeyer et al., 2022). The impact of snow and ice cover on phytoplankton light exposure can also be indirect. When ice is present but sufficiently clear, and any snow cover is sufficiently thin, light can penetrate and heat the waters just below the surface, which in turn can drive daytime convective mixing (Pernica et al., 2017). Convective mixing affects phytoplankton vertical positions and therefore average light exposures. Convective cells that are not too deep can keep cells suspended closer to the surface where they can obtain more light. This changes the competitive environment and can favour non-motile species that might otherwise sink out of the euphotic zone.

Phytoplankton growth is dependent on resource availability and physiology. Irradiance is (at any time of year) a critical parameter for phytoplankton, and it is of special interest during the winter, when seasonal decreases in incoming light and reduced penetration by virtue of snow and ice cover place strong limits on its availability. Photosynthesis—and therefore growth—is likewise limited, even when nutrients are sufficient. Nutrient sufficiency is often assumed for temperate lakes in winter, but there are exceptions, and in some cases this insufficiency will restrict growth. Temperate regions’ many oligotrophic lakes are expected to be nutrient limited in winter. Experimentation has provided evidence of winter nutrient limitation in at least one temperate mesotrophic lake: Knoll et al. (2024) supplemented nutrients during the winter in a Minnesota (US) lake and found evidence for a relief in nutrient limitation. Phytoplankton have been shown to require more nutrients as temperatures decrease (Rhee & Gotham, 1981). Photosynthesis is known to saturate at lower irradiances at lower temperatures (Edwards et al., 2016; Cavaliere et al., 2021), placing a maximum on growth rates that can only be overcome by increasing temperature. While at present these complex and poorly quantified relationships mean that we cannot easily produce accurate relationships between light availability and expected phytoplankton growth rates or community composition at cold winter temperatures (Edwards et al., 2016), we can still use empirical methods (like high-frequency sampling) to look for signs of net phytoplankton growth and responsiveness to increased irradiance under natural winter conditions.

Diatoms, which have high growth rates relative to other phytoplankton groups (e.g. MacIntyre et al., 2002), can respond quickly to increased irradiance at ice-off and can maintain under-ice seed populations (e.g. *Aulacoseira islandica* (O.Müller) Simonsen*,* Twiss et al., 2012; *Aulacoseira baicalensis* (K.I.Meyer) Simonsen, Katz et al., 2015). Researchers have reported a positive relationship between reduced ice-cover periods and increased diatom biomass (Twiss et al., 2012; Weyhenmeyer et al., 2013). Diatoms’ quick responses at ice-off may not be matched by changes in the timing of grazer abundances, and the resulting losses in grazer–prey synchrony can cause effects that ripple through the food web (Winder & Schindler, 2004; Adrian et al., 2006). Diatom abundances can also be affected by increased grazer overwintering survival in response to shortened periods between ice-on and ice-off (e.g. Hébert et al., 2021). The combination of available seed population, quick response time and (potentially) reduced grazing could be factors favouring diatoms in a changing climate. Observations of diatoms and their dynamics during the under-ice period itself, however, remain limited (Kong et al., 2021).

To explore the potential of in situ imaging technologies to study winter phytoplankton dynamics, we generated population-aggregated biovolume estimate time series for several diatoms and for total phytoplankton from samples collected with an *in situ* imaging-in-flow cytometer called the Imaging FlowCytobot (IFCB) in Lac Montjoie for the winter of 2014–2015. To our knowledge, this is the first deployment of an IFCB under lake ice. Diatoms were important contributors to winter biovolumes in our system, in addition to being of general interest by virtue of the malleability of the bloom timing of many taxa. We accompany our biovolume time series with measurements of the under-ice light environment and examine mixing conditions in the water column to interpret the observed patterns of biovolume change through time. We assess the IFCB instrument’s data collection strengths and weaknesses in the winter environment.

## Materials and methods

### Study site, moored profiler and IFCB

**Fig. 1:**
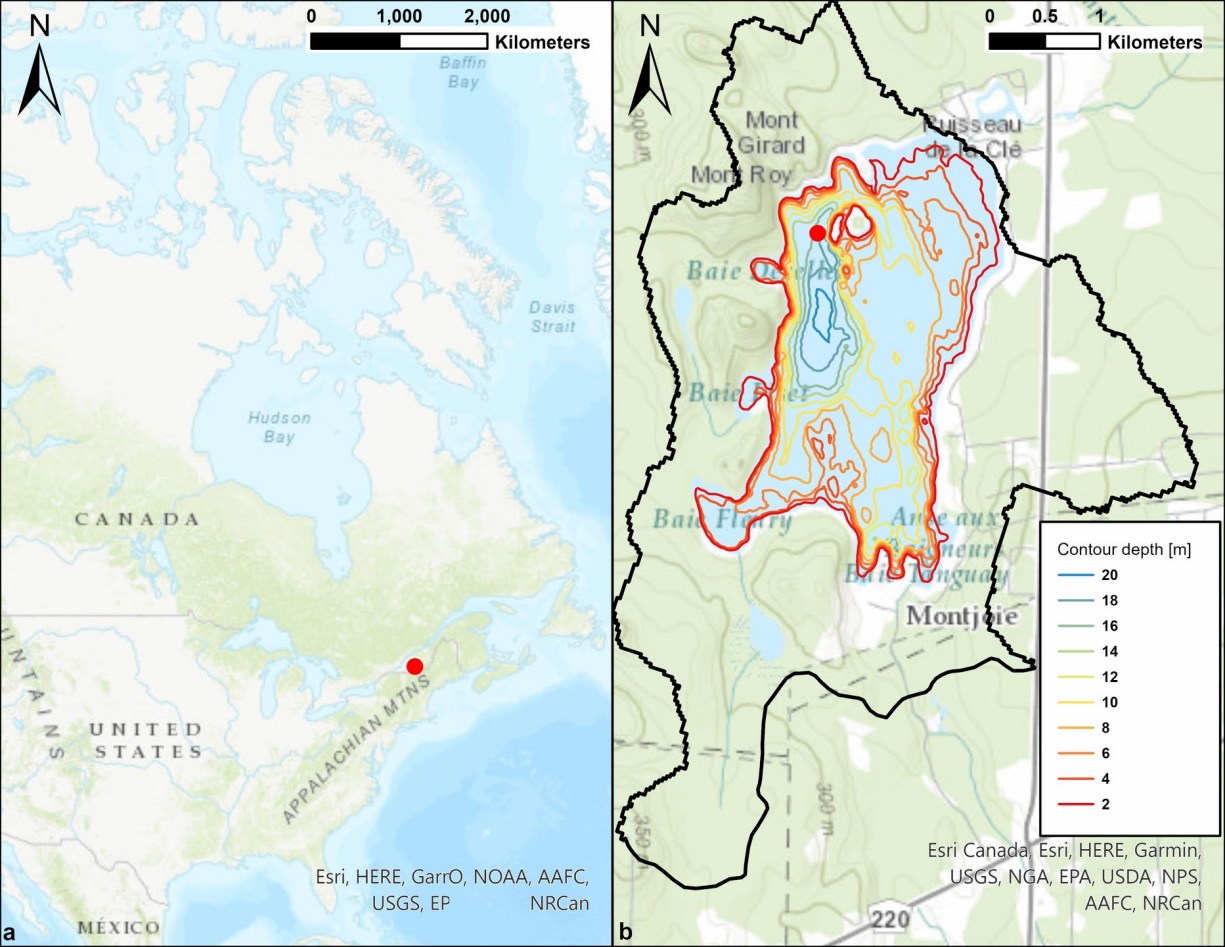
**a** Map of the IFCB profiling station’s location relative to North America and relative to (**b**) Lac Montjoie and its watershed (black line). The red dot is the location of the profiling station where the IFCB was located (45.4152043, − 72.1029829). The station was moored at a depth of 18 m near the deepest point in the lake. Image generated by Maxime Fradette

**Fig. 2:**
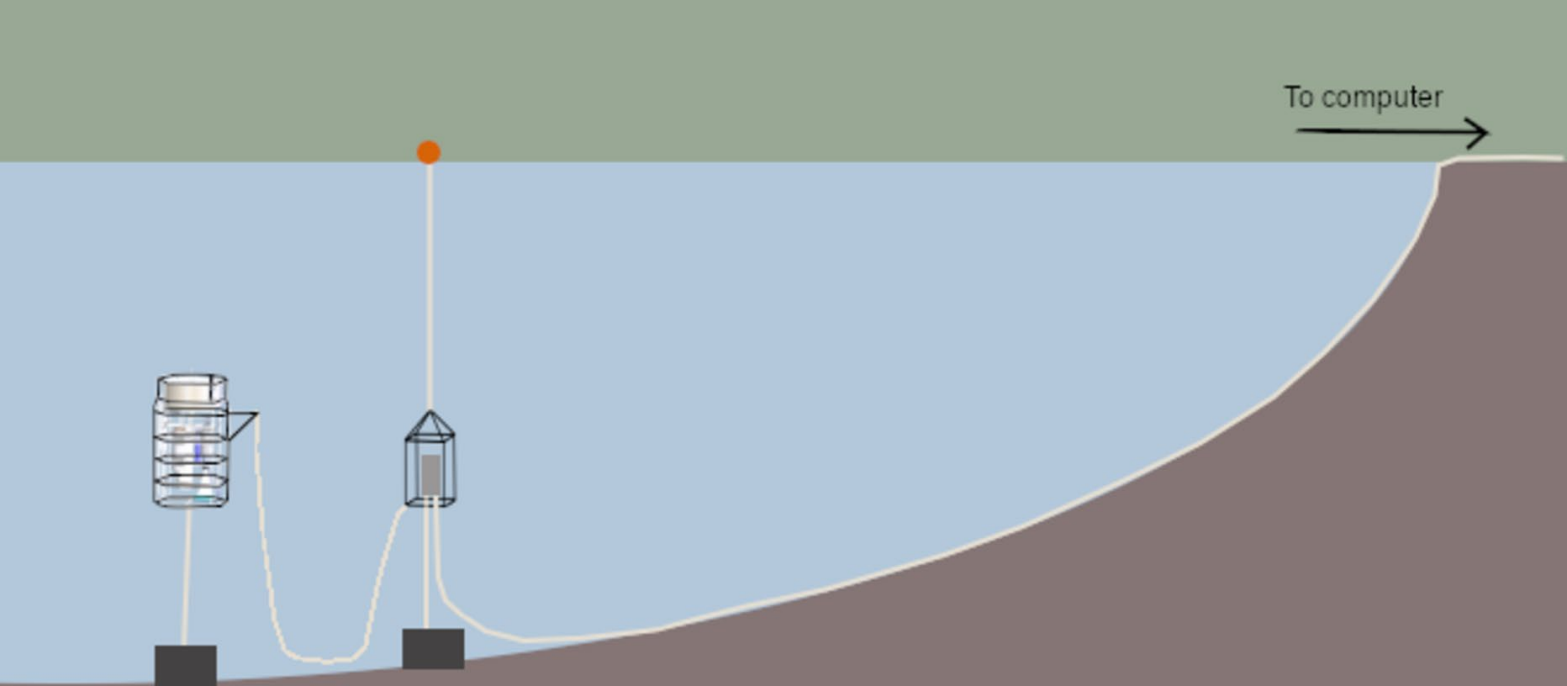
Schematic of moored profiling station. The AMP profiling cage anchored to the lake bottom and containing the IFCB, environmental instruments and winch are on the far left. They are cabled to a media converter, which is anchored to the lake bottom and connected to a float. The media converter allows the passage from a copper cable (which is more flexible and required for the profiling station) to a more fragile but high-performance fibre optic cable. The fibre optic cable extends to the shore along with the power, where it is connected to a cable situated under the deck of a home bordering the lake

An IFCB (Olson & Sosik, 2007) assembled at Woods Hole Oceanographic Institution (WHOI) was deployed mounted to an Autonomous Moored Profiler (AMP, WET Labs, USA) in mesotrophic lake Lac Montjoie, Quebec (Canada) (45.4092, − 72.0995; surface area 3.29 km^2^, maximum depth 22.2 m: Fig. 1). The AMP contained a winch whose cable was fixed to a heavy metal block on the lake floor. The AMP was connected to a media converter that was cabled to the shore (Fig. 2). Over the winter 2014/2015 sampling period, the winch positioned the IFCB to sample at 2, 4, 10 and 15 m depths. Lac Montjoie’s hydraulic features and watershed characteristics are detailed in Fradette (2016).

The IFCB used a 5-mL syringe to collect one water column sample for each ~ 25 min of operation. Samples were passed with a syringe pump (sample flow rate, 0.25 mL min^−1^; cell transit velocity through flow cell, 2.2 ms^−1^) into an imaging chamber. In the imaging chamber, particles encountered a laser that excited chlorophyll fluorescence. Grayscale images were collected from chlorophyll *a*-fluorescing particles with a minimum size of approximately 10 μm up to a maximum size of more than 100 μm (where the absolute upper limit was set by a 200-μm pore size intake filter). Sampling was concentrated at the shallower depths, with sampling frequencies approximately 5 times higher at the 2, 4, and 10 m depths than at the 15 m depth. We therefore consider only the 2, 4, and 10 m samples here. Between November 1, 2014, and April 30, 2015, 4,441 discrete water samples were obtained by the IFCB and included in our analysis.

### Environmental data collection

Instruments that measured the following environmental parameters were attached to the AMP’s metal cage alongside the IFCB: chlorophyll *a* fluorescence (WET Labs ECO-Triplet); hyperspectral downwelling irradiance, from which we used the 490 nm measurement (*E*_d_(490), Satlantic HyperOCR)—a proxy for photosynthetically utilisable radiation (PUR)—for our irradiance plots, and 398.76 nm through 700.04 nm—or approximately photosynthetically active radiation (PAR)—for our Richardson number calculations; temperature, conductivity, and depth (Seabird SBE19); and volume scattering function—a proxy for particle concentration—measured at 532 nm and 117° (WET Labs ECO Triplet). The winch was programmed to profile at times when IFCB samples were not being captured, since moving the instrument cage during an IFCB sample draw would have resulted in an IFCB sample that contained phytoplankton from multiple depths instead of the desired single depth. Profile frequency varied greatly (between 0 and 16 profiles/day, with a median of 10 from November through April, inclusive), as our winch functioned poorly at times. Profiles of environmental data were measured at a vertical spatial resolution finer than 10 cm. We pre-processed and quality-controlled the environmental data profiles. First, we removed bad profiles (e.g. empty files, files with unexpected depth recordings). Some of the *E*_d_(490) data contained gaps with depth: missing data were filled by fitting a linear model to the natural logarithm of the data and replacing missing data with the modelled result(s). All environmental data profiles were interpolated to address uneven sampling through time. A fine grid of depths (0 to 18.5 m, at 0.1-m intervals) versus times (at 20-min intervals) was used. The environmental data profiles were linearly interpolated over the grid to produce sections. The maximum interpolation interval was set to 6 h.

Additional data collected through traditional limnological sampling at low temporal frequency was made available via the Groupe de recherche interuniversitaire en limnologie (Interuniversity Research Group in Limnology, GRIL) “Lake Sentinels” program, which sampled Lac Montjoie from autumn 2012 through autumn 2015. Sample collection frequency varied with time of year: samples were collected monthly throughout the winters of 2013–2014 and 2014–2015 (except in December, when ice thickness was insufficient to support people sampling), and every two weeks at other times of year (Tapics et al., 2012). Discrete samples were collected from the epilimnion and metalimnion during stratified conditions, and from the mixed layer when stratification was not present. Ice and snow thickness was measured when present. Profiles of pH were generated with a YSI EXO1 probe.

### A directed, random-forest auto-classifier for IFCB image classification

We built a directed image auto-classifier using a random-forest algorithm in the IFCB-analysis software system, v.2 (Sosik et al., 2016). An image auto-classifier is software that considers an image’s data and—based on some criteria—assigns the image to a particular category (class). Alternately, it can decide that the image does not meet the criteria for assignment to any category and place it in a group called ‘unclassified’ or ‘other’. A *directed* image auto-classifier like ours must be provided a set of training data that it uses to learn how to distinguish the features of one category from another. (This contrasts with *undirected* auto-classifiers, which are provided no training data.)

### Classification based on feature data extracted from images

The auto-classifier that we built does not take images themselves as its input. Instead, it takes features extracted from images as its input. Features are single values (numbers) that represent different image characteristics and are extracted by performing various operations over an image. Metrics like shape (e.g. perimeter, area) and texture were amongst the several hundred features extracted from every IFCB image in our dataset. Feature extraction followed the methods of Sosik & Olson (2007), with method updates as documented in the IFCB analysis code repository (Sosik et al., 2016). The entire feature set is documented here: https://github.com/hsosik/ifcb-analysis/wiki/feature-file-documentation.

### Training data

We developed a large database of manually-classified IFCB phytoplankton images prior to this study. We selected images from this database, and their corresponding extracted features, to make our training set. The number of phytoplankton images available for each category in the database (e.g. *Asterionella, Synura)* was variable. Some phytoplankton are more abundant in Lac Montjoie than others, accounting for some of this variability. Some phytoplankton categories presented more varied morphologies than others (e.g. colonies), and we tried to include sufficient image numbers to capture that, which was another reason behind the variability in image numbers amongst phytoplankton categories.

### Selecting the phytoplankton categories of interest

The auto-classifier for this study was specifically designed to contain categories of phytoplankton that were present and contributed significantly to overall phytoplankton biovolume during the winter of 2014–2015. Auto-classification using a random-forest algorithm generally works best when there aren’t too many classes to be distinguished. For instance, a random-forest auto-classifier that must assign an image to one of 200 categories will tend to perform extremely poorly, whereas a random-forest auto-classifier that must assign an image to one of 20 categories has the potential to perform well (assuming the categories each have sufficiently distinguishable features). Random-forest classifiers also need a minimum amount of training data for each category to perform well. For this reason (and others), different auto-classifiers may be developed for different purposes. To get a better sense of what taxonomic groups might be of interest in Lac Montjoie during the winter of 2014–2015, we decided to look at biovolume estimates from our manually-classified verification samples. Verification samples were IFCB samples that had been randomly selected and then manually-classified by a human user to provide points of comparison with (and verification of) auto-classified samples, and are described in more detail under the heading ‘Verification samples’ (below). Biovolume estimates had previously been extracted from all IFCB images using the method described in Moberg & Sosik (2012). We created a histogram showing biovolume per mL for each group found in the manually-classified verification samples for winter 2014/2015. We then submitted high-winter-biovolume categories identified using the histogram to the algorithm that selected our classifier (described below). We also submitted the diatoms *Fragilaria* and *Tabellaria* to the algorithm: the histogram immediately suggested that diatoms should be included in the chosen categories, and despite being present in lower biovolumes, *Fragilaria* and *Tabellaria* were easily identifiable.

### Selecting the number of training images per category and number of trees

We needed to determine the optimal number of training images to submit to our random-forest classifier, but had no clear a priori reason to select a particular number. Random-forest classifiers work better when the number of training images provided for each category is somewhat balanced (i.e. there are roughly the same number of training images in each category). For some categories—particularly for small, common phytoplankton—thousands of training images were available in our training set database, while for other phytoplankton—larger and higher in biovolume per cell, but less common—there were often fewer available images. To obtain the best classifier possible, we had to identify the optimal number of training images to retain. Additionally, we had to decide whether to exclude certain of our pre-selected groups for lack of sufficient training images. Additionally, we needed to determine the optimal number of trees to grow in the random-forest step. Random-forest classifiers are built by creating several individual, independent decision trees. Each tree decides on its own what category to assign to an image. The category chosen by the most trees overall is the category that the random-forest classifier will assign to an image.

To identify the optimal number of training images and trees to include—that is, to try to develop an optimal classifier—we decided to run a systematic test where we varied the minimum image training set for a category to be between 200 and 500 images in steps of 100, and the maximum image training set to be between 300 and 900 in steps of 100. For each [minimum maximum] combination, we tested the result of growing 25 trees and the result of growing 50 trees. This resulted in 28 tested [minimum maximum] ranges and 2 numbers of trees, for a total of 56 tested classifier possibilities. At each loop, features extracted from the available training images were submitted to the IFCB software’s function ‘make_treeBaggerClassifier_iterative’, which included or excluded images from the offered set via selective iteration (program written and provided by Heidi M. Sosik, and adapted to our Mac operating environment). Twenty-four phytoplankton categories were submitted to this classifier training and selection algorithm. The category ‘Other’ contained cells whose image features were not sufficiently matched to any of the categories to make an assignment. ‘Other’ contained the highest biovolume overall, but as a catch-all category was excluded from training and selection. We selected the auto-classifier that produced the best results and then applied the trained auto-classifier to our winter period data to complete our automated counts.

### Verification samples

We tested how closely our auto-classified phytoplankton image counts matched the counts of a human assessor looking at the same digital image sample. The human assessor manually-classified three randomly selected image verification sample(s) for every two weeks of sampling. The time series of the auto-classifications versus the time series of the manually-classified verification samples for the phytoplankton categories of interest can be found in Supplemental 1.

### Density profiles and Richardson number

Convective mixed layers can form under lake ice when incoming solar radiation heats cold water near the surface, increasing temperature and decreasing density sufficiently to cause instability in the existing density gradient. Without sufficient solar radiative input, the water column under ice remains inversely stratified. To test for the presence of a convective mixed layer, we followed Pernica et al. (2017). An overview of the steps is provided here.

First, we generated water column density profiles using the limnology-specific LIM toolbox for MATLAB (described in Pawlowicz, 2008). We made carbonate estimates with LIM toolbox’s *limcarbonate*, using as inputs a single measurement of DIC from the Lake Pulse sampling program (Huot et el., 2019), and pH from a multiparameter sonde (EXO1, YSI, USA) that was mounted on the same cage as the IFCB. These and other chemical constituent data were then used to calculate lake-specific volumetric salinities in g L^−1^ using the LIM toolbox function *limcond*. The other chemical constituent data were measurements of calcium, magnesium, sodium, potassium, chloride, sulphate, ammonium, and nitrate from discrete epilimnion or mixed-layer samples from the Lake Sentinels program, and hydrogen ion and hydroxide ion estimates from pH profiles. The volumetric salinities (g L^−1^) were submitted along with temperature (°C) and pressure (decibar) profiles from the SBE19 Seabird CTD to the LIM toolbox function *limsal* to produce final density estimates (kg L^−1^).

We then determined density gradients with respect to depth and tested these gradients against a stability criterion (**∂ρ**/**∂z** < = 0.01 kg m^4^ at 0.5-m intervals) to determine whether there was a stable surface layer. If there was a stable surface layer, we calculated the Richardson number (R_*i*_).

We calculated the denominator of the Richardson number (the convective velocity at the convective test depth) using calculated solar radiation at depth, *Q* (W m^−2^), thermal expansibilities (*α*) and specific heat capacity (*c*_*p*_) as in Chen & Millero (1986). Unlike Pernica et al. (2017), we made our estimate of solar radiation at depth using planar irradiance profiles, PAR(z), from which we estimated scalar irradiance by applying a divisor of 0.75. We then calculated the numerator of the Richardson number (the buoyancy frequency) using the calculated density gradients. Finally, we combined the numerator and denominator to get the Richardson number. Then, we tested for the joint conditions that indicate that a convective mixed layer will form, *R* ≤ 1 and *b* > 0 (*α* < 0), where *b* (m^2^ s^−3^) is buoyancy flux.

## Results

### Environmental variables

**Fig. 3:**
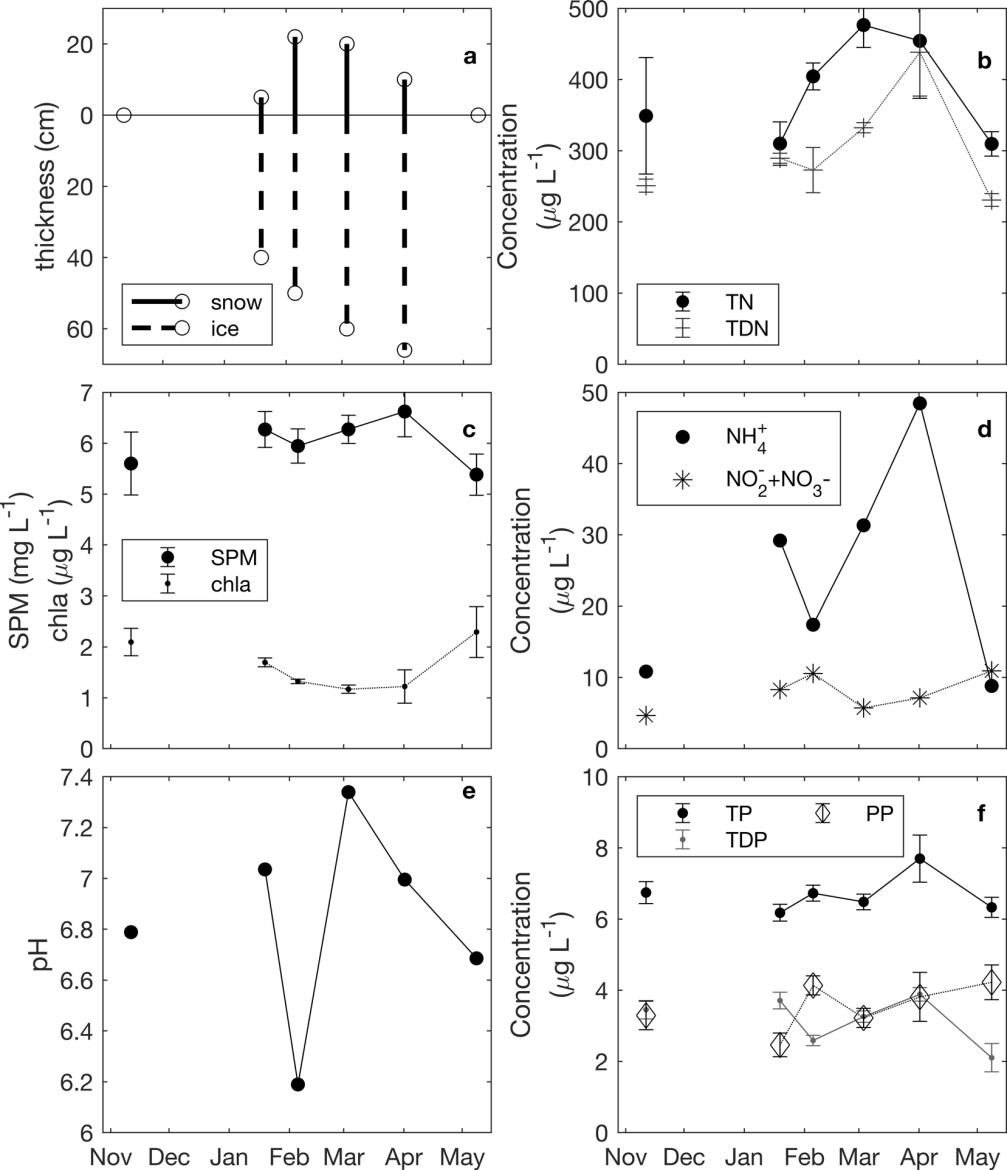
Environmental variables collected from November 2014 through May 15, 2015, during the GRIL Lake Sentinels sampling program. Acronyms for the discrete chemical samples are as follows: total nitrogen (TN), total dissolved nitrogen (TDN), suspended particulate matter (SPM), total phosphorous (TP), total dissolved phosphorous (TDP), and particulate phosphorous (PP). From November 11, 2014–April 1, 2015, samples were collected from a depth of approximately 0.5–1 m below the surface, and the lake showed no stratification or was moderately inversely stratified. Once the ice came off and the lake was strongly stratified (by May 8, 2015), the epilimnion was sampled at depths of 2 m and 4 m, and then, these were combined for analysis. No samples were taken for the month of December 2014. With the exceptions of ${\text{NH}}^{4+}$ and ${\text{NO}}_{2}^{-}+{\text{NO}}_{3}^{-}$, discrete chemical samples were taken in triplicate, rapidly processed and results averaged; the mean and sample standard deviation are presented here. ${\text{NH}}^{4+}$ and ${\text{NO}}_{2}^{-}+{\text{NO}}_{3}^{-}$ were each sampled once. Ice and snow thickness were each measured once near the auger sampling point. pH was calculated as the average of values taken within the sampled layer using the pH sensor of a YSI EXO1

Ice started forming in December but was too thin for sampling (Maxime Fradette, pers. comm.) (Fig. 3). Ice thickness showed a general increasing tendency from January 19 towards April 1, and reached a (measured) maximum of 66 cm. All the ice melted by May 8. Snow cover, unlike ice cover, did not show an increasing tendency from January through April. Instead, there were about 5 cm of snow on the ice on January 19, which increased to a maximum recorded snow thickness of 22 cm on February 5. Recorded snow cover was approximately the same at the March timepoint and fell to roughly half that by April, disappearing completely by May.

Total dissolved nitrogen (TDN) and total dissolved phosphorus (TDP) and ammonium (NH^4+^) all dropped between January 19 and February 5 and then increased towards April 1 before dropping again to measured minimums on May 8 (Fig. 3). Nitrite and Nitrate (${\text{NO}}_{2}^{-}$ + ${\text{NO}}_{3}^{-}$) varied less than NH^4+^, increasing from January 10 to February 5, then declining towards March and then increasing to May 8. Concentrations of phosphorus and nitrogen do not appear to be limiting for phytoplankton growth (Kim et al., 2022; Armin et al., 2023).

**Fig. 4:**
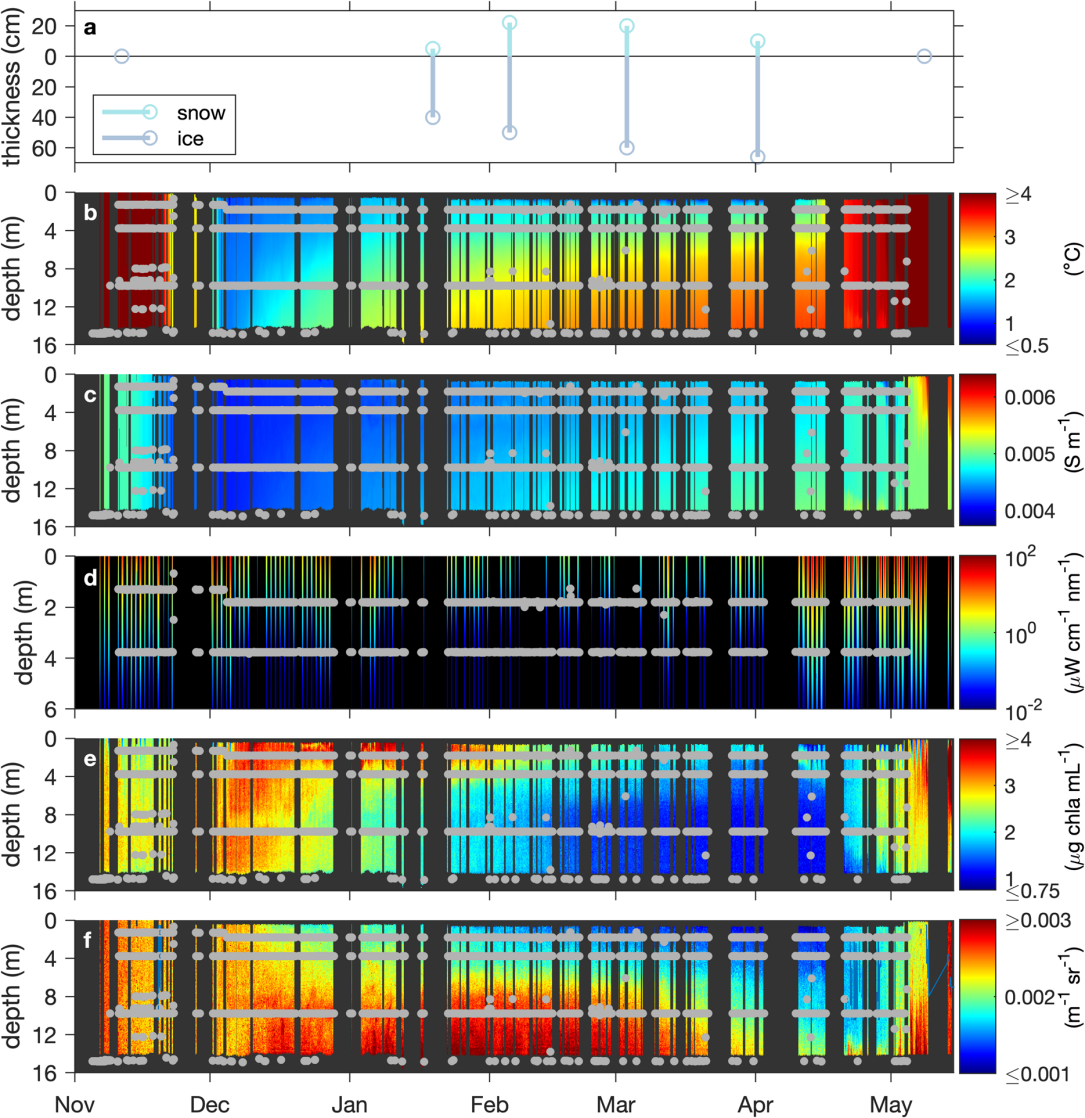
**a** Ice and snow thickness collected during the GRIL Lake Sentinels sampling program. **b** Temperature for winter 2014–2015 in Lac Montjoie. The colour bar range is restricted to from 0.5 °C to 4 °C, for improved visualization of temperature differences with depth in the later winter periods. Black indicates missing data. Temperatures above the maximum or below the minimum value of this range-restricted graph appear in the colour of the maximum or the minimum, respectively. The true range was 0.5 °C to 14.6 °C. Note that the maximum density of freshwater is at ~ 4.0 °C. Grey dots represent IFCB sampling points. **c** Specific conductivity for winter 2014–2015 in Lac Montjoie. Grey dots represent IFCB sampling points. Black indicates missing data. Units are Siemens m^−1^*.* **d** Irradiance at 490 nm (*E*_d_(490)) section for Lac Montjoie for the winter of 2014–2015. *E*_d_(490) has been transformed by taking its logarithm in base 10, for ease of visualisation. The colour bar range is given in the original units of *E*_d_(490) and has been restricted from 10^***–***2^ to 10^2^. Black indicates missing data or values that were zero prior to applying the log_10_ transformation. Grey dots represent IFCB sampling points. The true, untransformed range was 0–116.6 µW cm^−1^ nm^−1^. **e** Chlorophyll *a* fluorescence section for Lac Montjoie for the winter of 2014–2015. The colour bar range has been restricted from 0.75 to 4 to better permit visualization of fluorescence during the winter months. Black indicates missing data. Chlorophyll *a* fluorescence values above the maximum or below the minimum value of this range-restricted graph appear in the colour of the maximum or the minimum, respectively. Grey dots represent IFCB sampling points. The true range was 0.73 to 6.62 µg chla mL^−1^. Volume scattering function for winter 2014–2015 in Lac Montjoie. The colour bar range has been restricted from 0.001 to 0.003, to enhance small-scale differences. Black indicates missing data. Volume scattering function values above the maximum or below the minimum value of this range-restricted graph appear in the colour of the maximum or the minimum, respectively. Grey dots represent IFCB sampling points. The true range was 0.0008–0.006 m^−1^ sr^−1^

Temperature and specific conductivity were uniform across the water column until the beginning of December (Fig. 4b, c). As expected, temperatures increased from the surface towards the sediments during the winter period. There was a small general warming trend from December through to mid-April. Starting mid-December water was ~ 1 °C warmer close to the sediments (Fig. 4b). The water column temperature returned to being isothermal by mid-late April.

While irradiance levels (Fig. 4d) were relatively constant in November (despite day-to-day variability), it decreased sharply in early December then increased modestly in late December and early January. The lowest irradiances were observed in early February. Substantial increases in downwelling irradiance began again in early April.

Chlorophyll *a* concentration from in vivo fluorescence (Fig. 4e) was relatively uniform in the water column until the beginning of December. In December, a clear vertical structure of chlorophyll *a* concentration developed. First, we observed two broad maxima: one that spanned from the surface to approximately 8 m and another one between 10 and 14 m. As winter progressed, the surface maximum became shallower and stronger until end of January when the fluorescence decreased and remained low until the end of April. Towards the end of April and into May, we observed an increase in chlorophyll *a* concentration associated with the period of increased irradiance.

The volume scattering function is a proxy for particle concentration affected by all particles. Plots of the volume scattering function (Fig. 4f) show that particles were fairly evenly distributed with depth in November and early December and then became increasingly more common in deeper waters and less common in shallower waters as the winter progressed. Different dynamics can be seen by comparing the fluorescing (mostly phytoplankton) cells (Fig. 4e) with the total particle pool (Fig. 4f) during the winter months. Chlorophyll a fluorescence tends to be higher in shallower waters, while the total particle pool is generally more concentrated at depth, strongly suggesting a higher phytoplankton to non-algal particle ratio in surface waters. Assuming a constant quantum yield of fluorescence, this suggests two distinct particle populations, one with lower total concentration but a higher phytoplankton fraction at the surface and one with higher particle densities with lower phytoplankton fraction at depth.

We detected stable surface layers in Lac Montjoie over the interval from late January 2015 through early April 2015, but conditions for convective mixing under the stable surface layers were never met: the water column remained stable. It is possible that thin stable surface layers were also present prior to the end of January but went undetected because we typically did not have any density measurements above 1 m. When the first valid density point meets the instability criteria (in our case, the measurements at 1 m), no stable surface layer is diagnosed (Pernica & Baulch, 2017). Around January 22, 2015 we started to detect stable surface layers that were about 1.5 m thick. Around February 10, 2015 the detected layer deepened to 2 m. Near the end of February, it deepened again by another 0.5 m, and in the middle of March hovered around 3 m before shoaling and disappearing by mid-April. As a result of the depth variation in the stable surface layer the ‘2 m’, IFCB samples were sometimes below the stable surface layer and sometimes within it (see IFCB sample locations indicates as dots on the environmental data sections, Fig. 4b-f).

### Image classification

When selecting the phytoplankton categories to submit to the random-forest algorithm, we had looked to our manually-classified verification samples for the winter 2014–2015 period. We found that 59% (77 categories) of the 130 categories present in our training image database were not detected in the winter manually-classified verification set. The biovolume histogram shows that after the ‘Other’ category, four diatom taxa had the highest biovolumes: *Urosolenia* (previously known as *Rhizosolenia*)*, Asterionella*, Round diatoms and cf*. Synedra* (Supplemental 2).

The best-performing classifier had 500 to 700 images per category and grew 50 trees. It had a 13% overall error rate for accepted classifications and left 22% of images unclassified. Two diatom categories were excluded from the best-performing classifier due to these groups not achieving the 500 image threshold: unidentified Round diatoms and *Entomoneis.* The Round diatoms category had previously proven difficult to classify, as some cell orientations obscured identifiable features, and *Entomoneis* represented less than 5% of the biovolume of the cf. *Synedra* group in the winter verification samples. The diatom groups *Asterionella, Fragilaria, Urosolenia* and *conformis* (cf.) *Synedra* (the latter category containing small thin araphid diatoms) produced amongst the best results, while the auto-classification of the diatom *Tabellaria* performed less well. Classification results for the diatoms included in the classifier can be found in Supplemental 1. Sample images can be found in Supplemental 3.

The highly abundant and extremely fragile *Urosolenia* posed imaging problems for the IFCB. Its silica frustule was so thin and fine that our equipment frequently imaged only its chloroplasts, excluding the external structure (Supplemental 3). Since *Urosolenia* was often not imaged completely, we wanted to make sure that it was at least imaged consistently, so that increases and decreases in our biovolume time series would be reflective of changes in *Urosolenia* abundance, not changes in image capture. To ensure that changes in the IFCB imaging setup during deployment had not caused sudden changes in the distribution of estimated *Urosolenia* biovolumes, we created a time series of *Urosolenia* biovolume histograms. We found an increase in the number of very small objects (with estimated biovolumes less than 61 µm^3^) being classed as *Urosolenia*. We were able to link this increase to a change in the intensity of the IFCB’s flash lamp on February 5, 2015. Visual inspection revealed that the majority of these small images were *Urosolenia* chloroplasts. The small images were largely contained in a separate mode on the histograms. To improve the consistency of the time series, we ran an algorithm that removed these very small images over the entire winter period. As a result of these imaging problems, the *Urosolenia* biovolume estimates presented here are most certainly underestimates.

### Environmental and phytoplankton dynamics

**Fig. 5:**
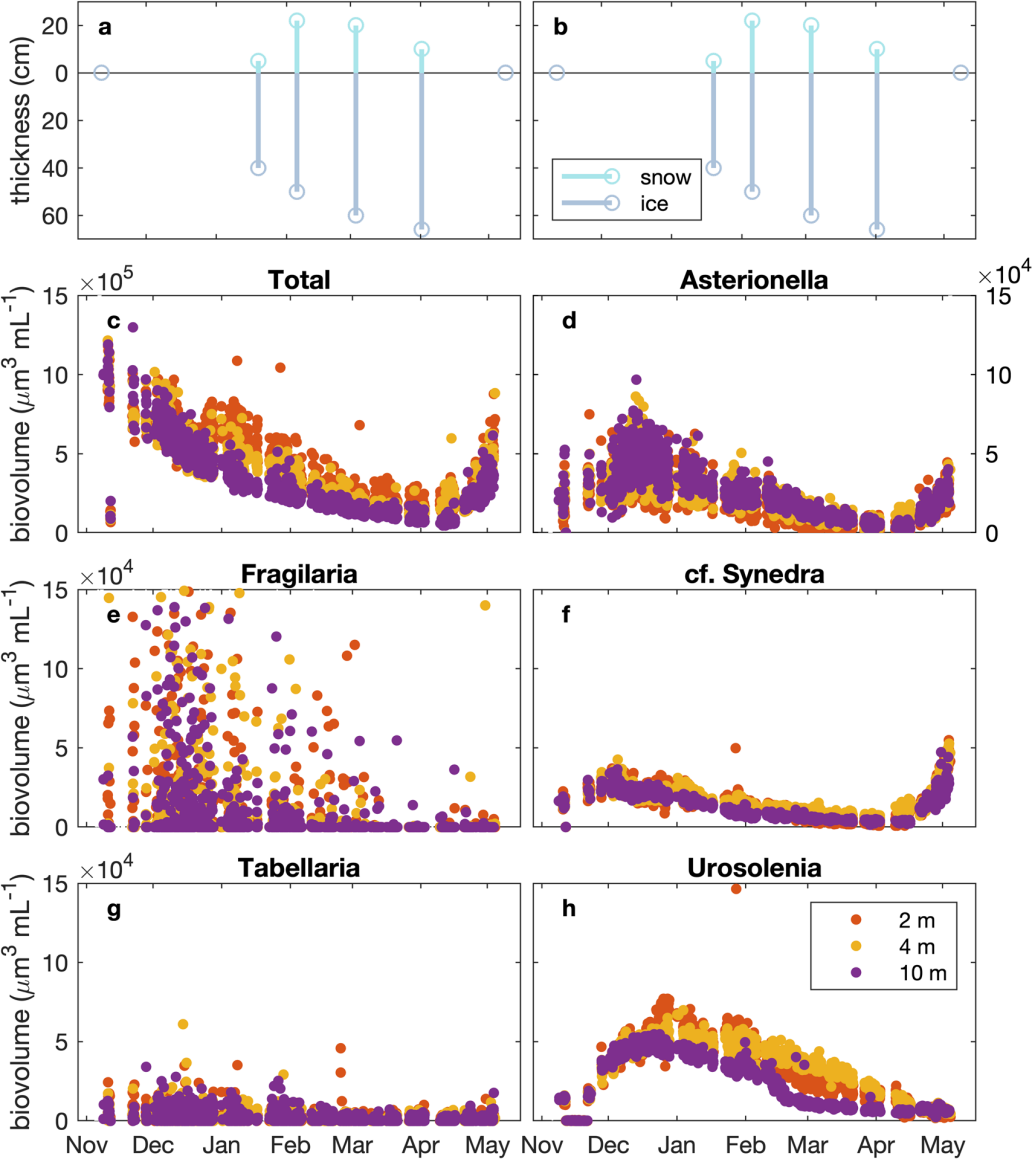
IFCB-generated biovolume estimates for chlorophyll a-fluorescing particles imaged during the winter of 2014–2015 in Lac Montjoie. **a,b** Ice and snow thickness, as measured during the GRIL Lake Sentinels sampling programme. **c** Total biovolumes per mL for imaged particles in each sample: note the factor of ten difference in the y-axis scale relative to the remaining panels, which represent different diatoms. **d** *Asterionella* biovolumes per mL. **e** *Fragilaria* biovolumes per mL. **f** cf. *Synedra* biovolumes per mL. **g** *Tabellaria* biovolumes per mL. **h** *Urosolenia* biovolumes per mL. Data for 15 m depth are not presented, as sampling frequency was far lower at this depth (roughly 2 samples for every 11 surface samples). For the interested reader, Supplemental 4 Fig. 1 presents the ± 12 h moving averages of this data

**Fig. 6:**
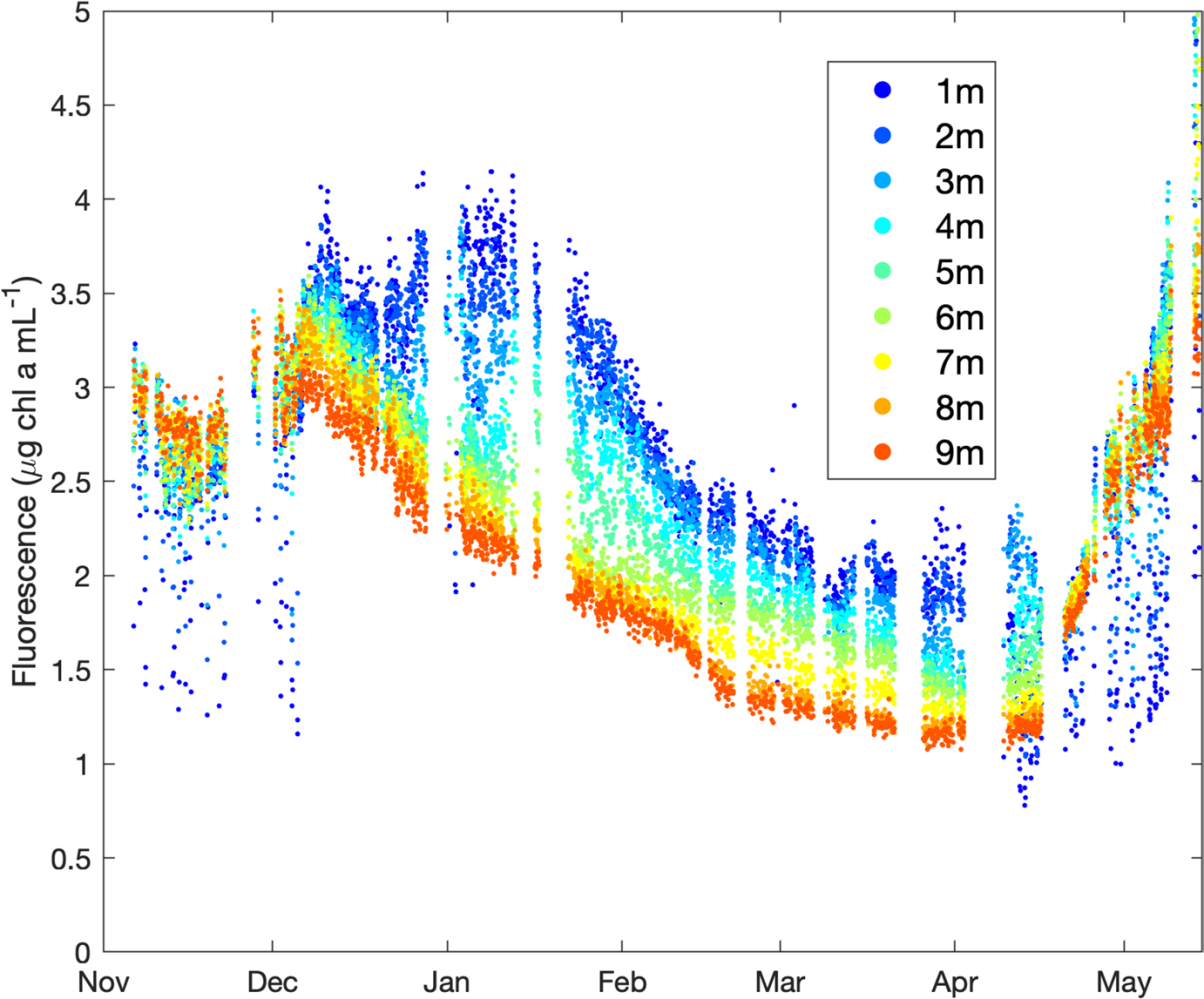
Chlorophyll a fluorescence through time as recorded in winter 2014–2015 at the Lac Montjoie profiling station and colour-coded by depth

Beginning in December, the total phytoplankton biovolume plot from the IFCB observations (Fig. 5c) shows a temporal pattern remarkably consistent with the plot of chlorophyll *a* fluorescence with magnitude colour-coded by depth (Fig. 6). Both illustrate a slight decline in phytoplankton towards mid-December, followed by a modest recovery towards early January at the surface, and then a long, slow decline until the onset of spring increases (the latter evident in the chlorophyll *a* time series).

The pattern of phytoplankton biovolume with depth was relatively consistent: the quantity of phytoplankton was highest at the surface and declined with depth for Total biovolume (Fig. 5c), *Synedra*-like (Fig. 5f) and *Urosolenia* (Fig. 5h) (but not *Asterionella*, Fig. 5d). Early January showed recovery at the surface only, while at depth phytoplankton quantities decreased. *Urosolenia* showed distinct differences in biovolume with depth, with highest biovolumes per mL occurring in the better-lit surface waters (Fig. 5h). At the surface, the small mid-December increase in light (Fig. 4d) seems to have permitted growth, and biovolume estimates only show substantial declines in February. At 10 m, the biomass decreases more rapidly starting at the beginning of December. *Asterionella* abundances increased during the cold season, with biovolume estimates trending upwards until mid-December, then falling slowly towards spring (Fig. 5d). Overall, *Asterionella* maintained around 15% of its December-peak value (~ 8 × 10^4^ µm^3^ mL^−1^) through to the spring. However, note that the *Asterionella* signal is quite noisy, perhaps owing to their varying colony sizes. Unlike *Urosolenia*, *Asterionella* showed little difference in abundance over depth, but did show an earlier decline over the winter. Cf. *Synedra* diatoms, similar to *Urosolenia*, recovered somewhat with the mid-December through mid-January light increase at the surface (Fig. 5h). The *Fragilaria* time series was quite noisy, but nonetheless showed an overall decline towards spring (Fig. 5e). Noise in this series can be almost entirely attributed to the very low colony counts – *Fragilaria* was not one of the high-biovolume taxa identified in our histogram, but was added in because it was a highly identifiable diatom. Examples of absolute colony counts (not normalized to biovolume) can be seen in the Supplemental verification plots, S1.Fig. 1. *Tabellaria* (Fig. 5g) was generally poorly classified and is not discussed further here.

## Discussion

### Interpretation of the results

Successful overwintering sets the starting point for a species’ productivity in the upcoming year(s) (e.g. Sommer et al., 2012; Cirés et al., 2013; Fang & Sommer, 2017). Overwintering is not well quantified, particularly in freshwater systems, yet is central to understanding different taxa’s seasonal and interannual dynamics (Maberly et al., 1994). Diatoms are of particular interest, as it has been suggested that increasing winter light availability associated with decreasing snow and ice cover may offer them a competitive advantage. Our winter observations of the diatoms *Asterionella,* cf. *Synedra,* and *Urosolenia*, made by taking advantage of the strengths of a moored IFCB, provide what we think to be the first high-frequency observations of changes to these seed populations under ice in a temperate lake.

The patterns of phytoplankton abundance that we observed at three depths from the late autumn through the onset of spring in Lac Montjoie were in some ways expected, and in other ways surprising. As outlined in the literature, we saw evidence that diatoms could take advantage of near-surface light levels to maintain, or even grow, their populations during the winter period (Fig. 5).

The observed pattern in the total winter biovolumes of chlorophyll a-fluorescing particles also supports the idea that increases in light under-ice can be associated with phytoplankton growth, particularly near the surface (Fig. 5c). Incident irradiance at the surface is affected by atmospheric conditions, like cloudiness, and seasonal changes. Snow cover (and to a lesser extent ice thickness) influences penetration of irradiance into the water column. Underwater, optical characteristics of the water column like scattering and absorption play important roles in light transmission (Massicotte et al., 2019; Hazuková et al., 2021). We had in situ irradiance measurements and consider how the observed patterns in irradiance may have related to measured ice and snow depths. The ice and snow depth data were taken at a very low temporal resolution compared to the irradiance data and will not have captured rapid depth fluctuations, but give a good indication of trend over the measured period. Temperatures above zero that started on December 23 and continued until December 28, 2014 likely caused snowmelt and increased light penetration at this time. The lowest irradiances were observed in early February. Substantial spring increases in downwelling irradiance would seem to coincide with the disappearance of snow and ice (see top left panel of Fig. 2, which shows that the heavy ice cover and moderate snow cover present at the beginning of April 2015 has disappeared by early May 2015). However, based on our observations, not all the studied diatoms seem to have benefitted equally or responded in the same way. These differences could influence the competitiveness and survival of different taxa as they encounter changing ice and snow cover under conditions of climate change. *Asterionella* maintained the same population trends across all three measured depths, and although it maintained considerable biovolumes through to the spring, it began its decline earlier than the other observed taxa. No gains related to the increase in light were evident in the early January samples, contrasting with the other taxa. Likewise, *Asterionella* was the only diatom whose biovolumes consistently increased with depth. *Asterionella* is recognized as an abundant bloom-forming genus and was one of two diatom groups from this study that were seen to rapidly respond to the onset of spring conditions (see Supplemental 4 for a version of Fig. 5 to which a moving average has been applied and which makes this increase even more visible). *Asterionella, Fragilaria* and *Synedra* have all been previously identified as common spring-bloom-forming species (Werner, 1977; Agbeti & Smol, 1995). The long and thin cf. *Synedra* appear to have benefitted from the increase in surface light towards the end of December, resulting in a small recovery in biovolume. They maintained around 20% of their December-peak value (~ 3.5 × 10^4^ µm^3^ mL^−1^) through to spring. The cf. *Synedra* population maintained was sufficient to rapidly return them to December-peak values at the onset of spring conditions. This responsiveness to increasing light suggests that *Synedra* might be better suited than *Asterionella* to advantage from any increases in snow melt that might be associated with global warming. *Urosolenia,* like cf. *Synedra* diatoms, seem to have benefitted from the increase in surface light, as evidenced by a recovery of biovolume towards the end of December. Unlike for spring-blooming *Asterionella* and cf*. Synedra* diatoms, however, the winter decline in *Urosolenia* was not followed by a rebound at the onset of spring. Previous studies have shown that *Urosolenia* do well in low light environments, which may be why it did relatively well in winter. The increasing frequency of rain events compare to snow events forecasted with climate change will likely provide another source of changing light environment that may favour species such as *Urosolenia* that benefit from short term increases in irradiance (Musselman et al., 2018). Lac Montjoie was stably stratified throughout the analysed ice-cover period, and the lack of convective mixing may have been another factor favouring the relatively high biovolumes maintained by slow-sinking *Urosolenia* under ice.

Our study took place over one winter during which Lac Montjoie experienced snow and ice cover thick enough to prevent the formation of convection cells. Even under these conditions we evidenced net under-ice growth of *Asterionella*, cf. *Synedra* and *Urosolenia* when light was sufficient. We expect that Lac Montjoie, as other temperate lakes, will experience decreasing snow and ice cover in the future and possibly even period of open water. Increasing convection and light availability will likely not only change the competitive environment to favour non-motile taxa like the diatoms, but also change competition between diatoms over winter. The low-light adapted, easily suspended *Urosolenia* may lose the advantages that made it competitive in the winter of 2014/2015, and be displaced by heavier, light-responsive bloomers like *Asterionella* and cf. *Synedra.* How low water temperatures may temper maximum growth rates and affect this competition is unknown, as are the water temperatures we can expect in the future.

### The pros and cons of automated phytoplankton counting

The IFCB proved highly useful for studying winter season diatom dynamics, giving a unique high-temporal resolution look at an under-ice lake environment. The IFCB can process many hundreds of times more samples than could be processed manually and render accessible sampling conditions that can be otherwise difficult or dangerous (e.g. thin-ice conditions that are very difficult to sample, storms, etc.). Even with concerted effort, limited numbers of samples can be collected and processed by hand. For example, the discrete depth-resolved winter sampling work by Chiapella et al. (2021) studying phytoplankton by microscopy was limited to 7 timepoints over 24 h. A potential downside of relying on the IFCB—or any piece of deployed equipment—is that there is always the possibility of breakdown. When this occurs under-ice, instrument recovery and re-deployment are unlikely to occur until spring thaw, and the planned winter data collection will be incomplete. However, this risk is reasonably small for an instrument that is maintained well prior to deployment and is outweighed by the benefits.

There are some limitations associated with the IFCB’s digital phytoplankton samples when compared to microscopy samples. The IFCB cannot provide the same taxonomic resolution as a carefully performed microscope count. The grayscale images of the IFCB limit the identification and separation of phytoplankton that have common shapes (like sphere) but distinctive pigments. Nor are the biovolume estimates extracted from individual IFCB images expected to be as accurate as those obtained by microscope. A nuance of the IFCB’s biovolume estimates is that they are calculated for each image, and an image may contain a single cell or a colony. The biovolume estimated for the cell or entire colony is reported as the biovolume for the image. By contrast, microscopists typically work at the unit of the cell and often do not record colony sizes. The IFCB cannot provide a biovolume per cell, because it does not count the individual cells in a colony. However, the IFCB provides information on population size distribution for colonial taxa that would not be provided by a typical microscopy count. The IFCB’s biovolume estimates can also be somewhat affected if other organisms (e.g. fungi, parasites) are attached to the phytoplankton being analysed, as the algorithm calculating the biovolumes may include these organisms when outlining a phytoplankton’s perimeter. An advantage of the IFCB biovolume estimates is that they never contain the type of calculation errors that can be associated with manual biovolume calculations (errors which, in our experience, can be both occasional or [if a calculation has been misunderstood] long-term). The IFCB is more limited than the microscopist in terms of the size range of phytoplankton that it can assess. A microscopist can switch lenses or techniques to count organisms from microns through hundreds of microns in size, while an IFCB’s size range is fixed. IFCB samples do not degrade and analysing them is not a destructive process, differentiating them from preservative-fixed samples. They can be re-assessed if new questions arise or if more powerful classification techniques become available. Of course, like any type of sample, IFCB samples/data must be carefully stored to preserve their integrity (i.e. backed up to avoid data damage or corruption). Consistency is a distinct advantage of algorithm-based classification systems like those applied to the IFCB’s data. Unlike microscopists, classification algorithms never fatigue or produce inconsistent opinions on sample content. But the robotic regularity of the IFCB is only a benefit when the user commits to maintaining data quality. Consistent results require consistent image quality, and obtaining consistent images requires a certain vigilance on the part of the IFCB operator, who must use the system’s software to remotely monitor for changes in image focus and other parameters. The usefulness of an assessment undertaken with IFCB data depends on how well the applied classifier works, and current techniques have limitations (for instance, the number of categories that can be classified at once). Working in the favour of automated platforms, advances in techniques like artificial intelligence will likely continue to improve image classifications in the near future.

There are time and financial costs associated with operating an automated platform. A clear downside of the IFCB is its initial cost (plus maintenance costs and field personnel). The labour associated with using automated instruments can be similar to the effort associated with a typical field campaign of the same length. Developing training data sets to establish the auto-classifiers required to sort and count the collected images requires considerable time and taxonomic expertise, paralleling the need for time and expertise required when undertaking microscopy counts. Likewise, manually classifying verification samples for comparison with the IFCB’s automated output is a best practice and requires similar effort to that required to count microscope samples. In our case, we manually-classified three full samples for every two weeks’ collected data during our work: this is slightly more effort than might be expended on counting a single winter sample per week under a microscope (although the total data obtained in the IFCB case is much greater). Automated platforms are not hands-off or labour-free, but are an investment with long-term benefits. They are tools that can help us unravel the mysteries of the winter season, if in designing our research questions we carefully consider the instrument’s limitations and strengths.

*Urosolenia* presented the greatest challenge to accurate quantification, as the delicate frustule was frequently truncated by the algorithm designed to rapidly delineate phytoplankton and isolate them from the background during imaging in the IFCB. However, conventional bottle sampling methods which employ Lugol’s solution as a sample fixative can destroy the fine frustule of *Urosolenia*, which can be difficult to see under a microscope even when intact (Tremarin et al., 2013). Large underestimates can reasonably be expected for manual counts. Based on these findings, it seems that the IFCB offers advantages in the winter sampling of *Urosolenia* even when (as here) the software does not capture the images optimally. For example, the high temporal and spatial frequency resolution allowed us to show that *Urosolenia* showed a strong variation in abundance with depth. *Urosolenia*’s exceptionally thin, light, high surface-area body likely keeps it suspended in more favourable light environments far better than denser, more compact taxa. Similarly, we found that *Urosolenia* made a significant contribution to the overall phytoplankton winter biovolume (at times ~ 10% of Total biovolume) even though the IFCB system likely underestimated the *Urosolenia* biovolume.

Given the great challenges associated with sampling the under-ice environment, the importance of taking advantage of available technologies is evident. Future winter IFCB work in Lac Montjoie might benefit from specific instrument calibration to improve the clean capture of *Urosolenia* images, providing greater confidence in the estimates of *Urosolenia* biovolumes. Likewise, future continuous winter-through-spring deployments would be useful to capture the effect of under-ice seed population maintenance on spring blooms. Finally, collecting zooplankton data to inform the interpretation of loss rates would also provide a more complete picture of the lake’s under-ice ecology (Hébert et al., 2021).

## Conclusion

To our knowledge, this is the first deployment of an in situ flow cytometer in an ice-covered lake over winter. Our vertically resolved, high-frequency time series provide a novel perspective on under-ice diatom dynamics. This study of Lac Montjoie confirms that winter is far from a period of stasis for phytoplankton, and the capacity to respond quickly to short periods of increased light may be a determining factor that favours the seed population of one species over another. Further advances in classification techniques (e.g. Kraft et al., 2022; Orenstein et al., 2022) and instrumentation will likely improve our ability to implement finer taxonomic studies of phytoplankton from this kind of platform.

## Supplementary Information

Below is the link to the electronic supplementary material.

Supplementary file1 (DOCX 855 KB)

Supplementary file2 (DOCX 95 KB)

Supplementary file3 (DOCX 5118 KB)

Supplementary file4 (DOCX 2492 KB)

Supplementary file5 (CSV 475 KB)

## Data Availability

The sampling time, depth, sample volume, phytoplankton class, and estimated biovolume (µm^3^) are provided for the classes of interest over the studied winter period as a.csv file. Sentinels data are archived in a Borealis data repository with the identifier 10.5683/SP3/AIDIGQ. The MATLAB routines for analysis of data from the Imaging FlowCytobot (IFCB) are available at https://github.com/hsosik/ifcb-analysis.
