# Supplementary material for: High-frequency diatom dynamics seen in an ice- and snow-covered temperate lake using an imaging-in-flow cytometer
Source: Hydrobiologia. 2025 Feb 6;852(11):2887–905. doi: 10.1007/s10750-025-05802-8 (PMC11982112; doi:10.1007/s10750-025-05802-8)
Supplement: Supplementary file 1 — Supplementary file1 (DOCX 855 KB) [file 10750_2025_5802_MOESM1_ESM.docx]

**Time series of the auto-classifications versus the manual classifications for the IFCB verification samples**


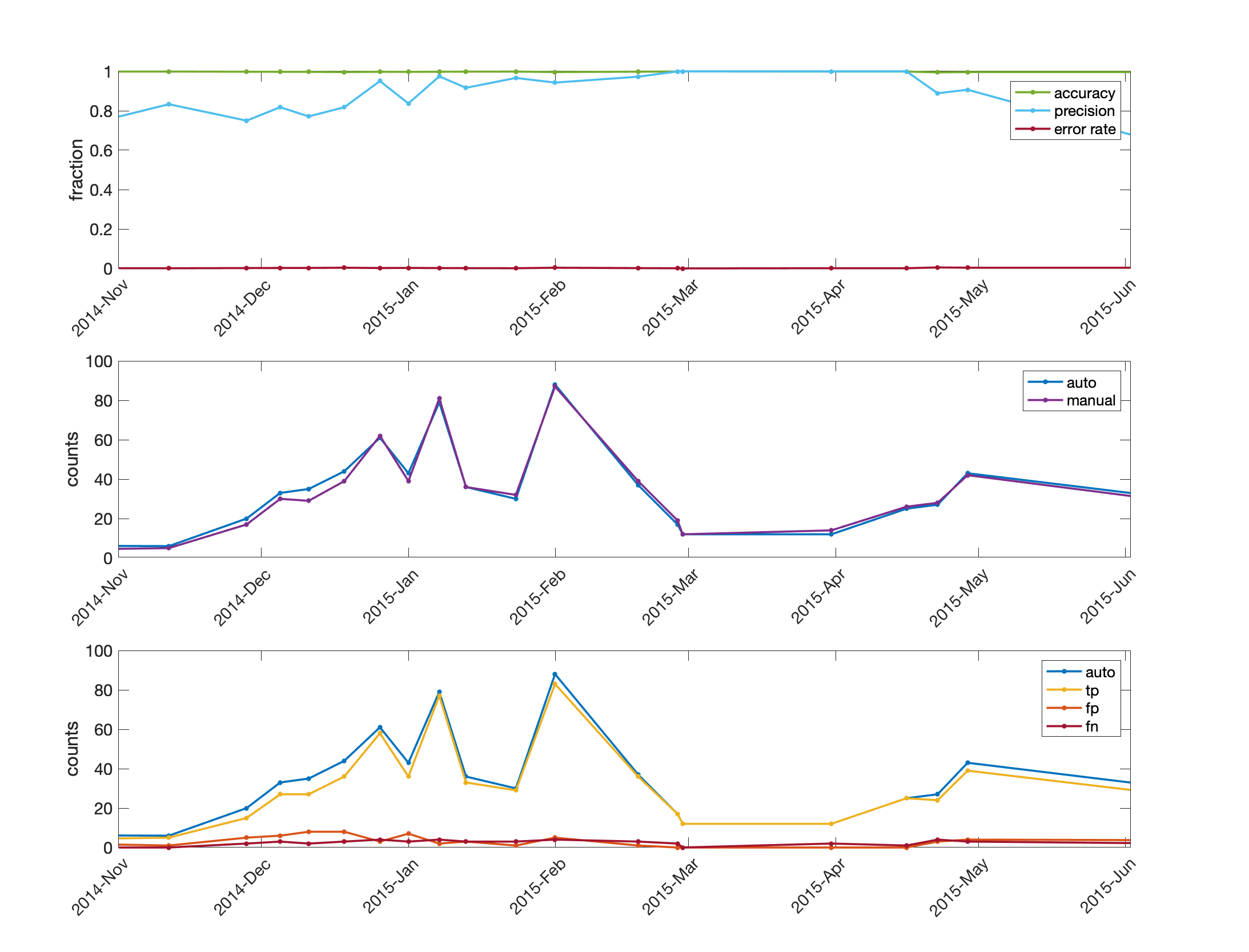


**S1.Figure 1**.Assessment metrics for autoclassification of *Asterionella* for winter 2014-2015 and beyond. Top panel: accuracy, error, and precision based on comparison of autoclassified and manual verification samples. Middle panel: Counts of images classified as *Asterionella* by a human user (manual verification samples) and the autoclassifier (auto). Bottom panel: Counts of autoclassified *Asterionella* images that were true positives (tp), false positives (fp), and false negatives (fn). True negatives (tn) are the (thousands of) non-*Asterionella* that were correctly excluded from the *Asterionella* category, and are not plotted here.


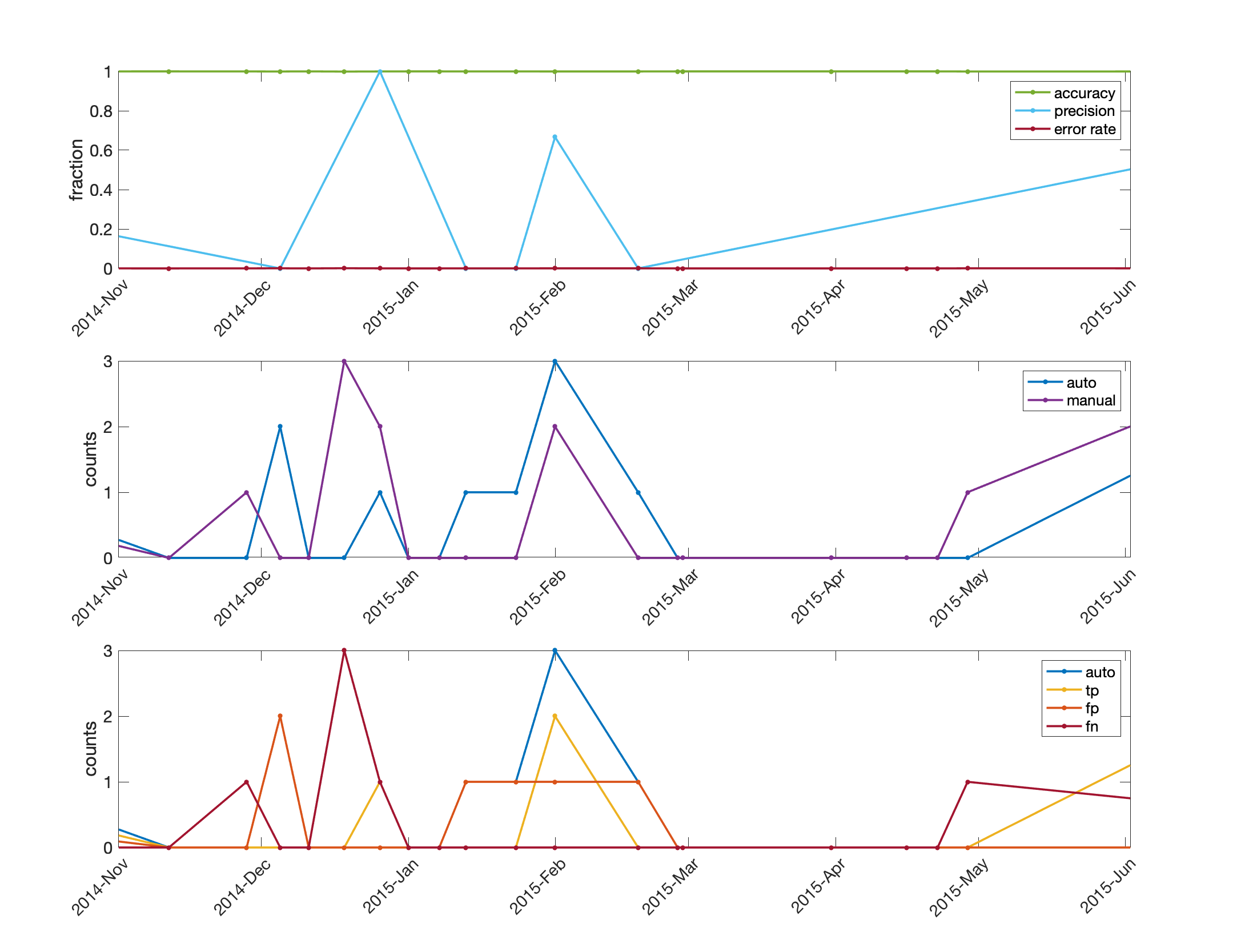


**S1.Figure 2**.Assessment metrics for autoclassification of *Fragilaria* for winter 2014-2015 and beyond. Top panel: accuracy, error, and precision based on comparison of autoclassified and verification samples. Middle panel: Counts of images classified as *Fragilaria* by a human user (manual verification samples) and the autoclassifier (auto). Bottom panel: Counts of autoclassified *Fragilaria* images that were true positives (tp), false positives (fp), and false negatives (fn). True negatives (tn) are the (thousands of) non- *Fragilaria* that were correctly excluded from the *Fragilaria* category, and are not plotted here.


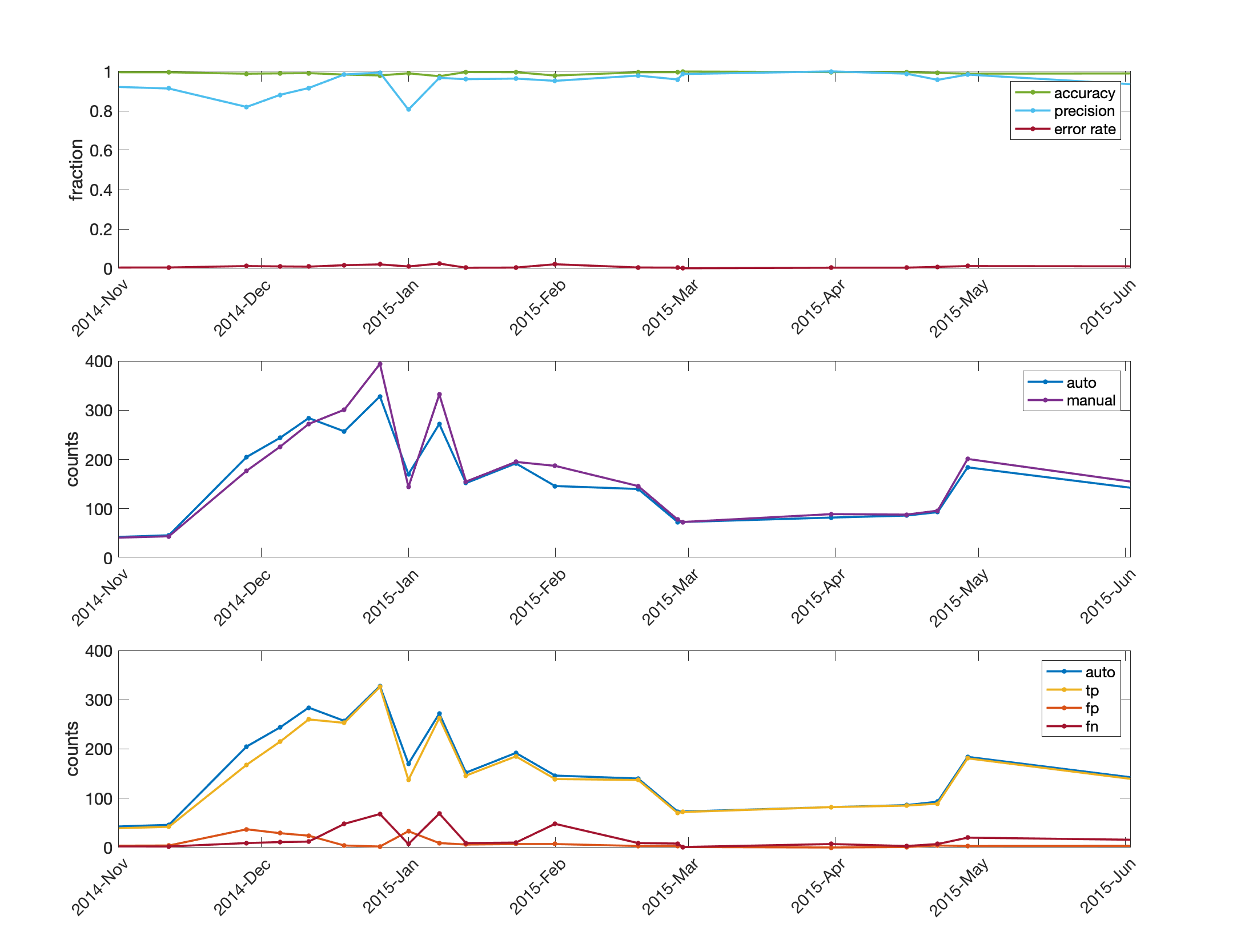


**S1.Figure 3**.Assessment metrics for autoclassification of *cf. Synedra* for winter 2014-2015 and beyond. Top panel: accuracy, error, and precision based on comparison of autoclassified and verification samples. Middle panel: Counts of images classified as *cf. Synedra* by a human user (manual verification samples) and the autoclassifier (auto). Bottom panel: Counts of autoclassified *cf. Synedra* images that were true positives (tp), false positives (fp), and false negatives (fn). True negatives (tn) are the (thousands of) non- *cf. Synedra* that were correctly excluded from the *cf. Synedra* category, and are not plotted here.

**
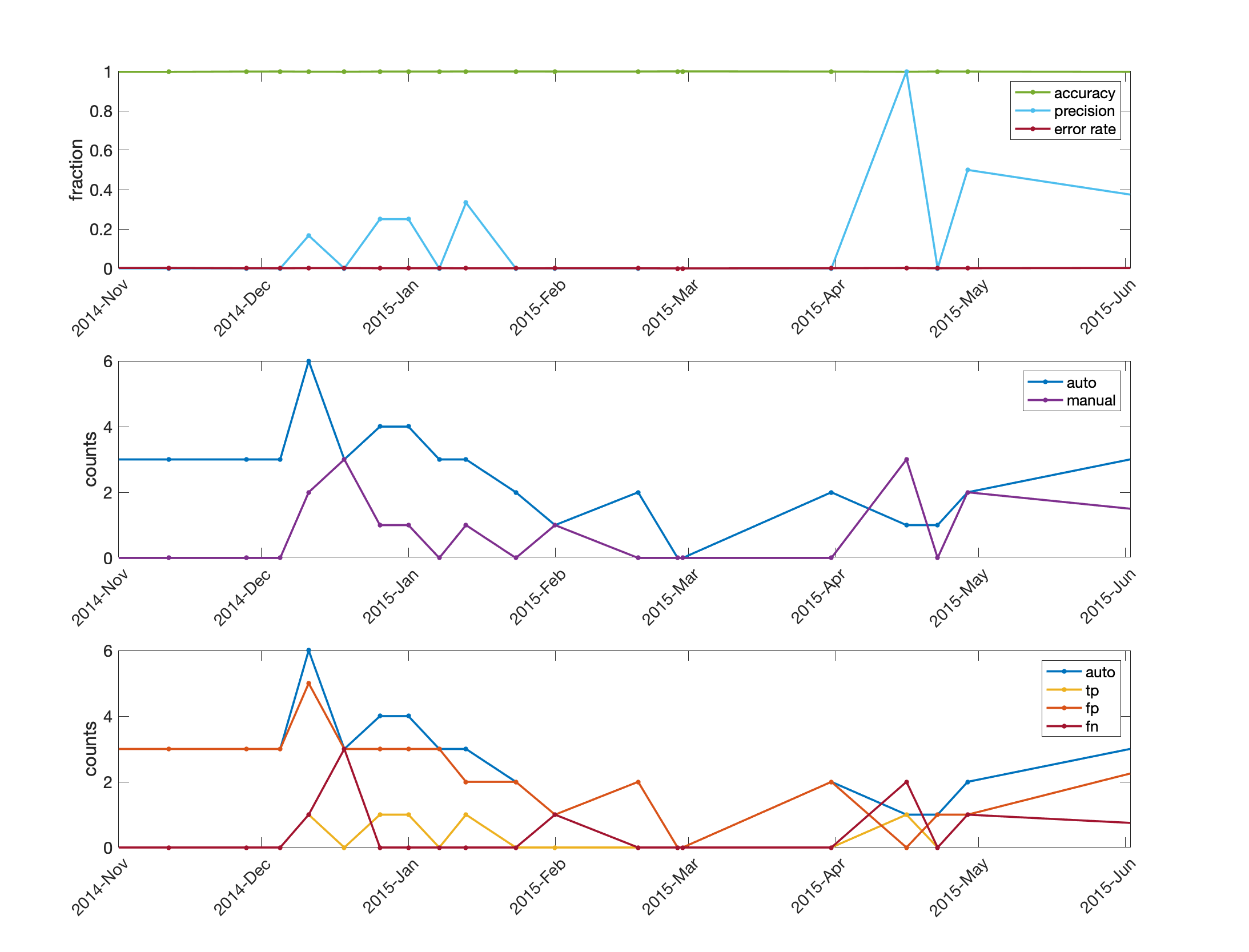
**

**S1.Figure 4**.Assessment metrics for autoclassification of *Tabellaria* for winter 2014-2015 and beyond. Top panel: accuracy, error, and precision based on comparison of autoclassified and verification samples. Middle panel: Counts of images classified as *Tabellaria* by a human user (manual verification samples) and the autoclassifier (auto). Bottom panel: Counts of autoclassified *Tabellaria* images that were true positives (tp), false positives (fp), and false negatives (fn). True negatives (tn) are the (thousands of) non-*Tabellaria* that were correctly excluded from the *Tabellaria* category, and are not plotted here.

**
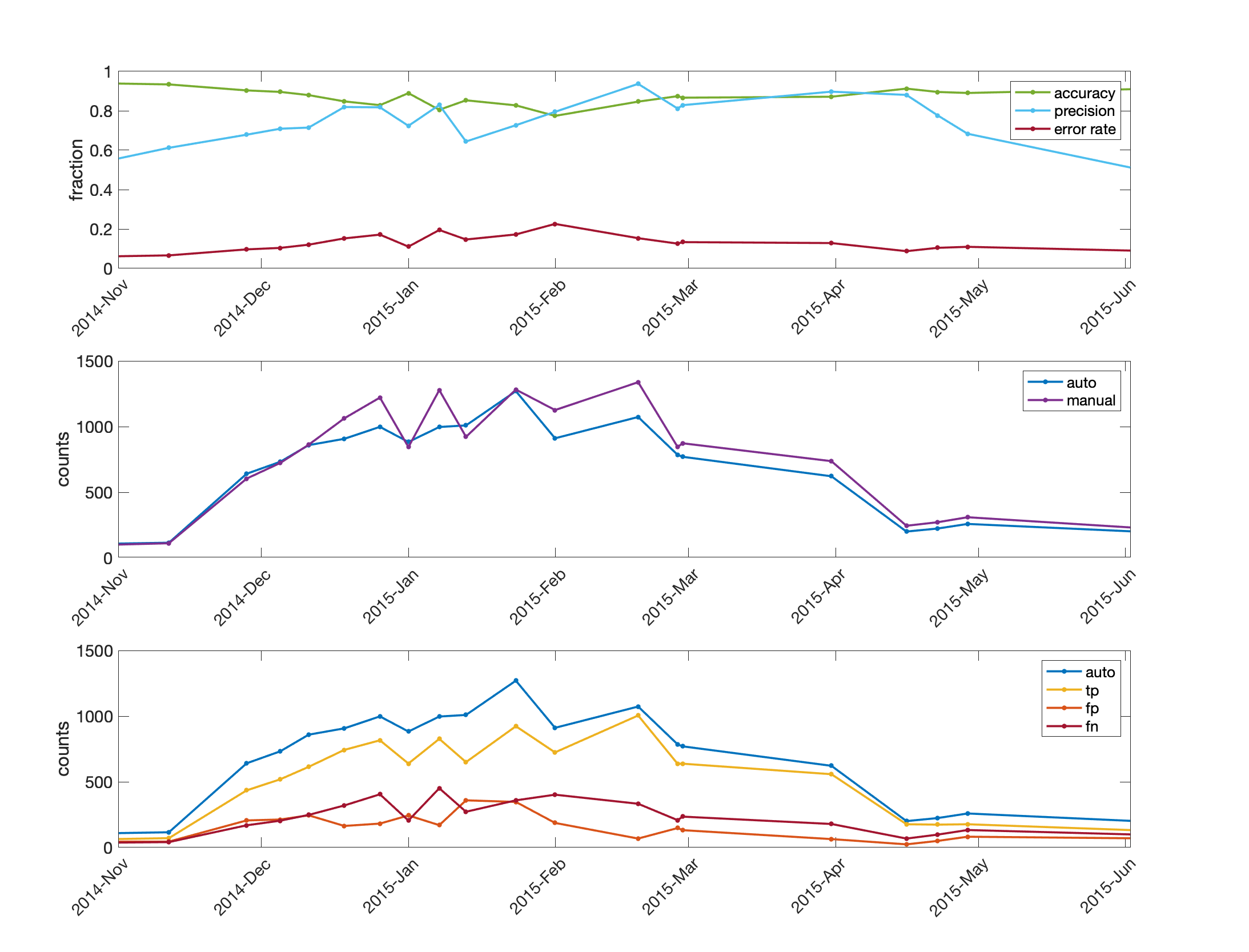
**

**S1.Figure 5**.Assessment metrics for autoclassification of *Urosolenia* for winter 2014-2015 and beyond. Top panel: accuracy, error, and precision based on comparison of autoclassified and verification samples. Middle panel: Counts of images classified as *Urosolenia* by a human user (manual verification samples) and the autoclassifier (auto). Bottom panel: Counts of autoclassified *Urosolenia* images that were true positives (tp), false positives (fp), and false negatives (fn). True negatives (tn) are the (thousands of) non-*Urosolenia* that were correctly excluded from the *Urosolenia* category, and are not plotted here.
