# Supplementary material for: High-frequency diatom dynamics seen in an ice- and snow-covered temperate lake using an imaging-in-flow cytometer
Source: Hydrobiologia. 2025 Feb 6;852(11):2887–905. doi: 10.1007/s10750-025-05802-8 (PMC11982112; doi:10.1007/s10750-025-05802-8)
Supplement: Supplementary file 2 — Supplementary file2 (DOCX 95 KB) [file 10750_2025_5802_MOESM2_ESM.docx]

**
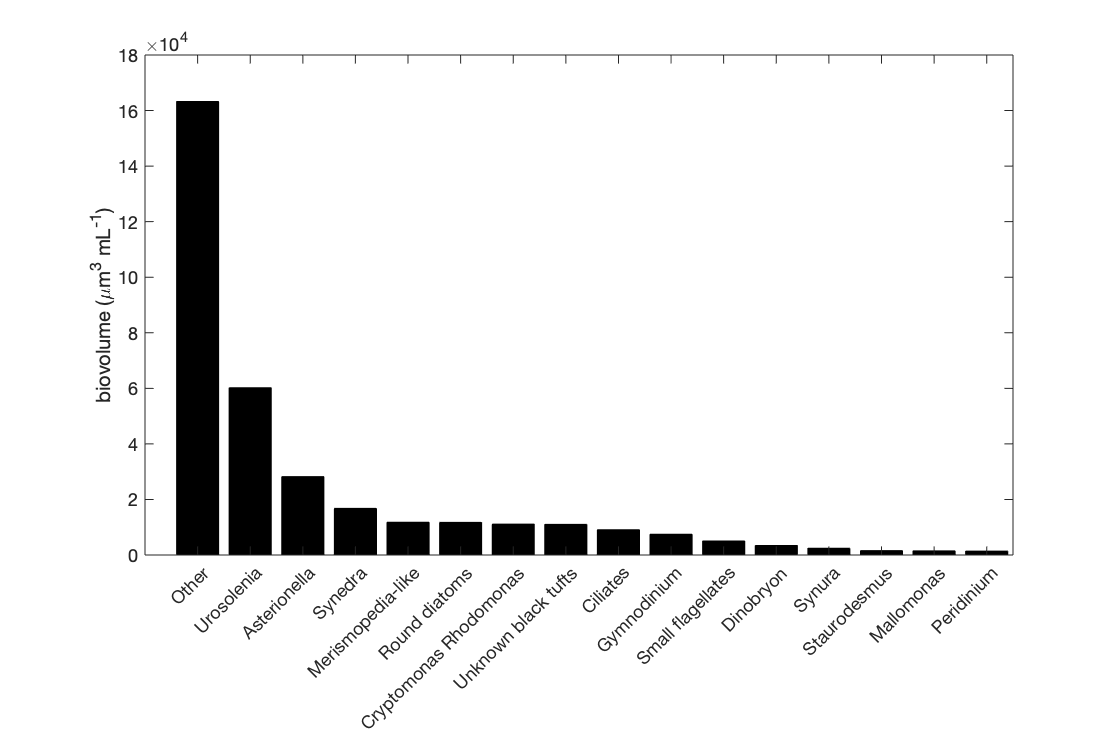
**

**S2.Figure 1** Biovolumes of the top fifteen taxonomic groups in the manual classification set summed for the winter period of November 2014 through to the middle of April 2015. We include the category of unknowns, ‘Other’, to provide information on the unclassifiable fraction in the set. Since the manual classification set is not evenly distributed in terms of time of day/depth, this is only a qualitative distribution and may not represent exactly the true winter proportions. The high diatom biovolumes drove our interest in that group, and we included some that did not appear in the top fifteen (e.g., Fragilaria, Tabellaria) in our subsequent analyses.
