# Supplementary material for: High-frequency diatom dynamics seen in an ice- and snow-covered temperate lake using an imaging-in-flow cytometer
Source: Hydrobiologia. 2025 Feb 6;852(11):2887–905. doi: 10.1007/s10750-025-05802-8 (PMC11982112; doi:10.1007/s10750-025-05802-8)
Supplement: Supplementary file 3 — Supplementary file3 (DOCX 5118 KB) [file 10750_2025_5802_MOESM3_ESM.docx]

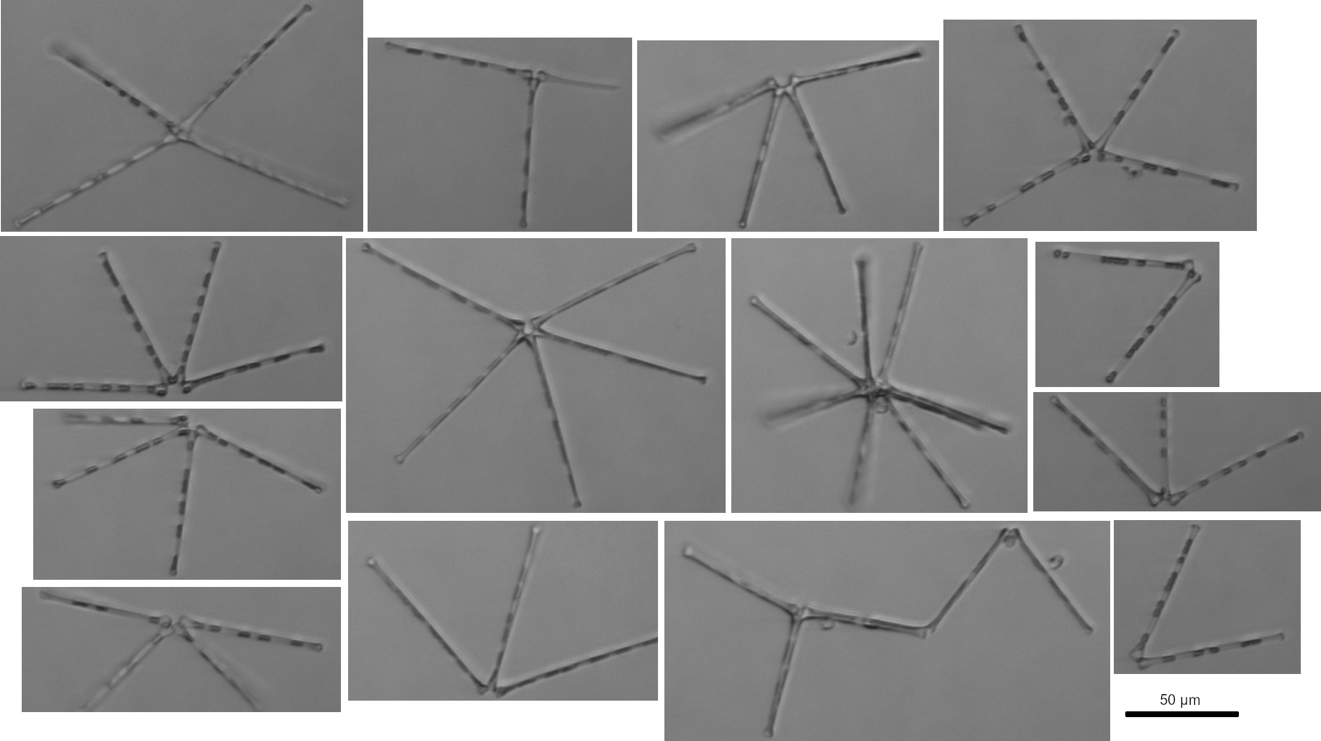


S3.Figure 1 *Asterionella* images from the IFCB.


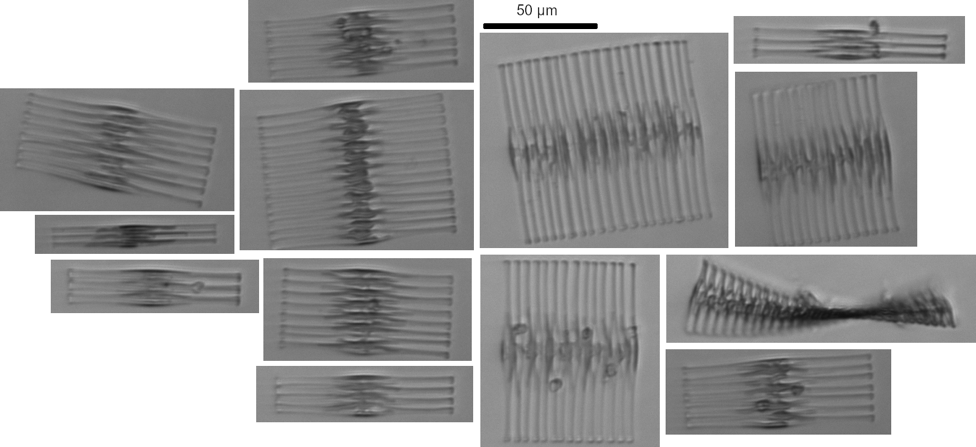


S3.Figure 2 *Fragilaria* images from the IFCB.

*
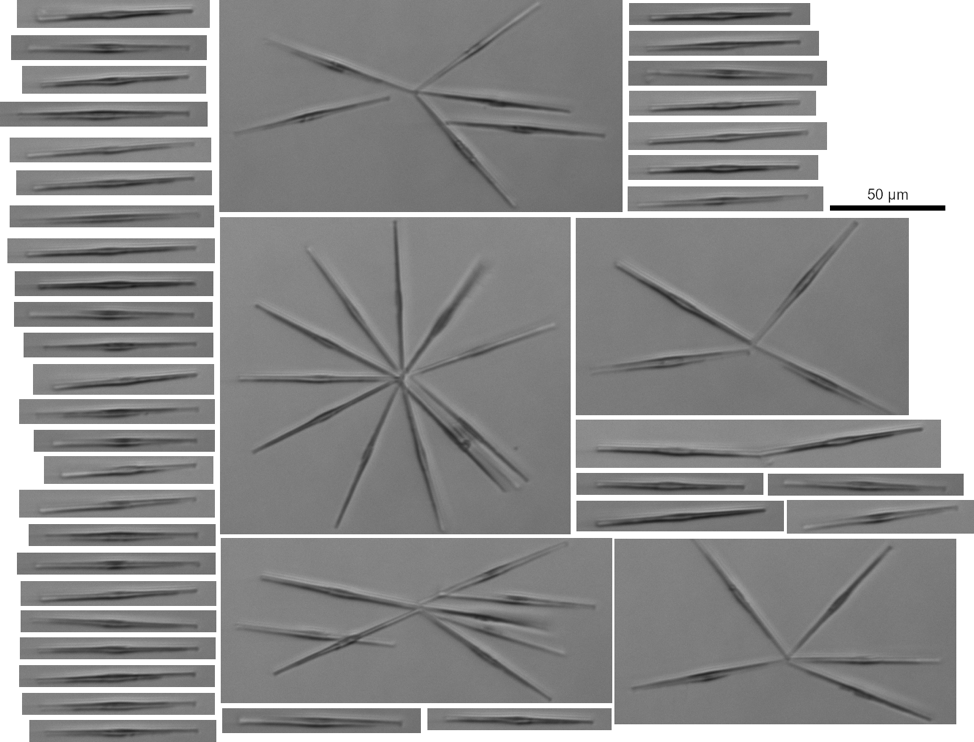
*

S3.Figure 3 cf. *Synedra* images from the IFCB.


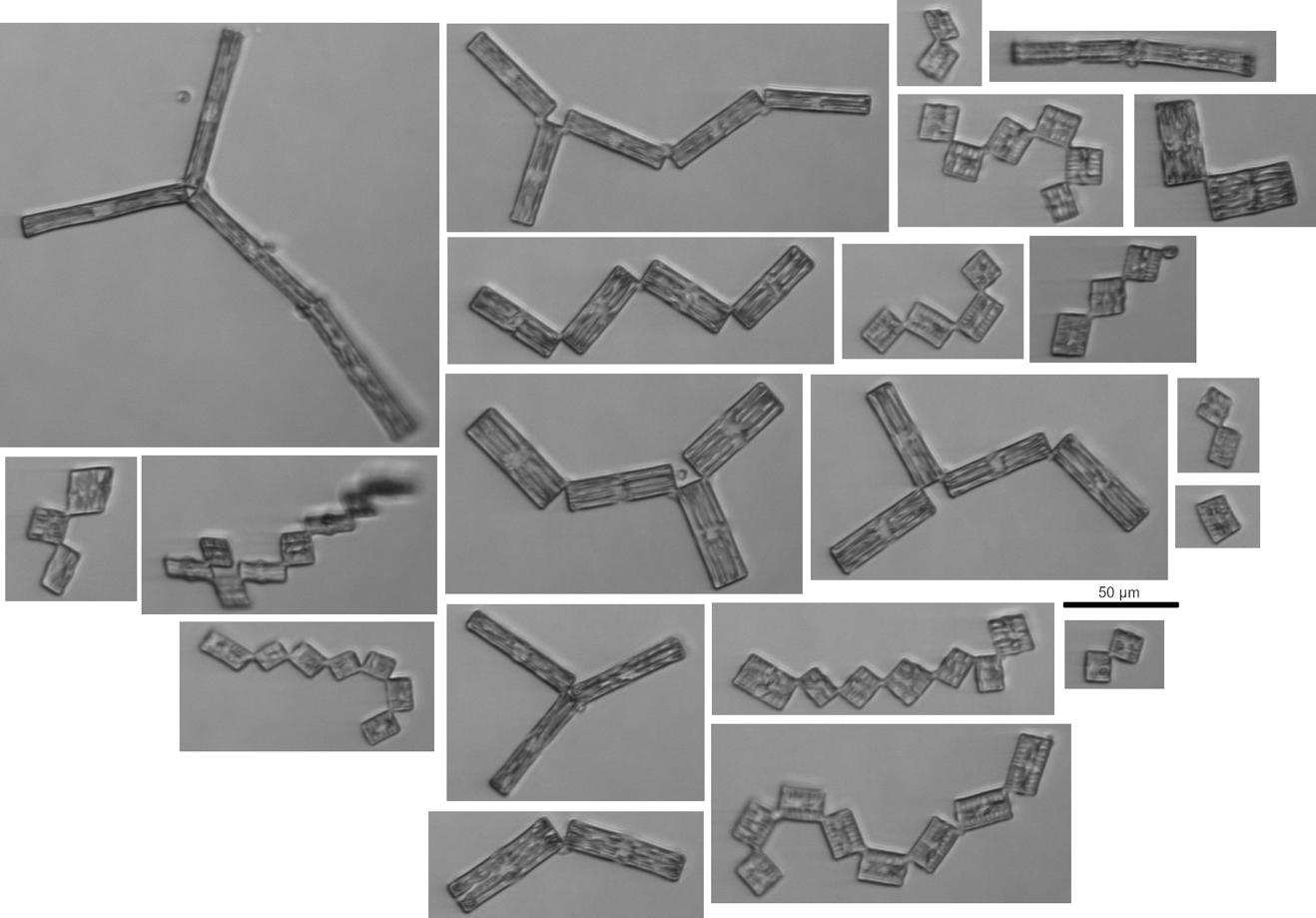


S3.Figure 4 *Tabellaria* images from the IFCB.

*
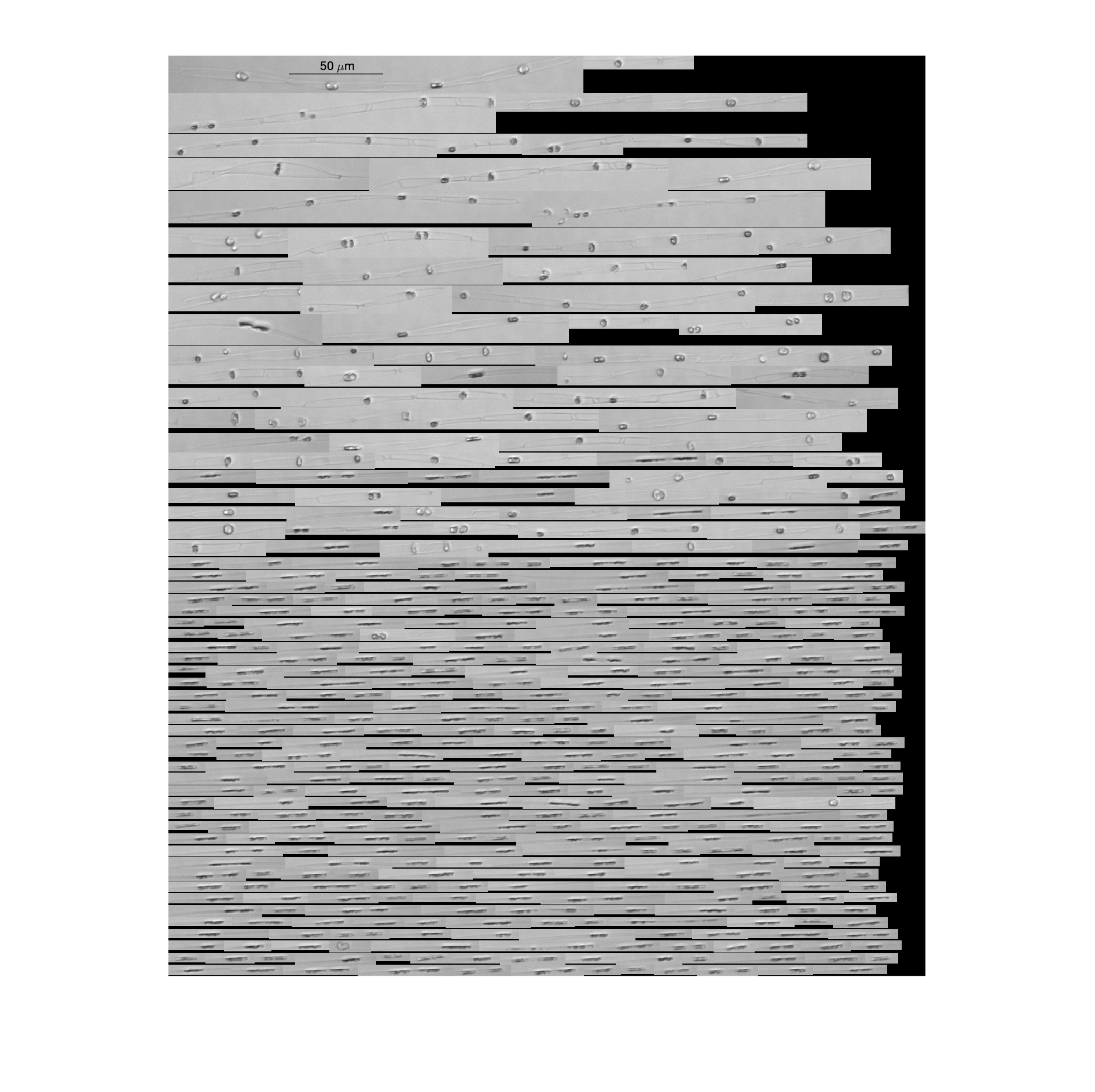
*

S3.Figure 5 *Urosolenia* images from the IFCB. In the upper images, *Urosolenia* was largely captured, and in the bottom images, *Urosolenia* was heavily cropped.
