# Supplementary material for: High-frequency diatom dynamics seen in an ice- and snow-covered temperate lake using an imaging-in-flow cytometer
Source: Hydrobiologia. 2025 Feb 6;852(11):2887–905. doi: 10.1007/s10750-025-05802-8 (PMC11982112; doi:10.1007/s10750-025-05802-8)
Supplement: Supplementary file 4 — Supplementary file4 (DOCX 2492 KB) [file 10750_2025_5802_MOESM4_ESM.docx]

**Moving average of the IFCB winter diatoms time series**


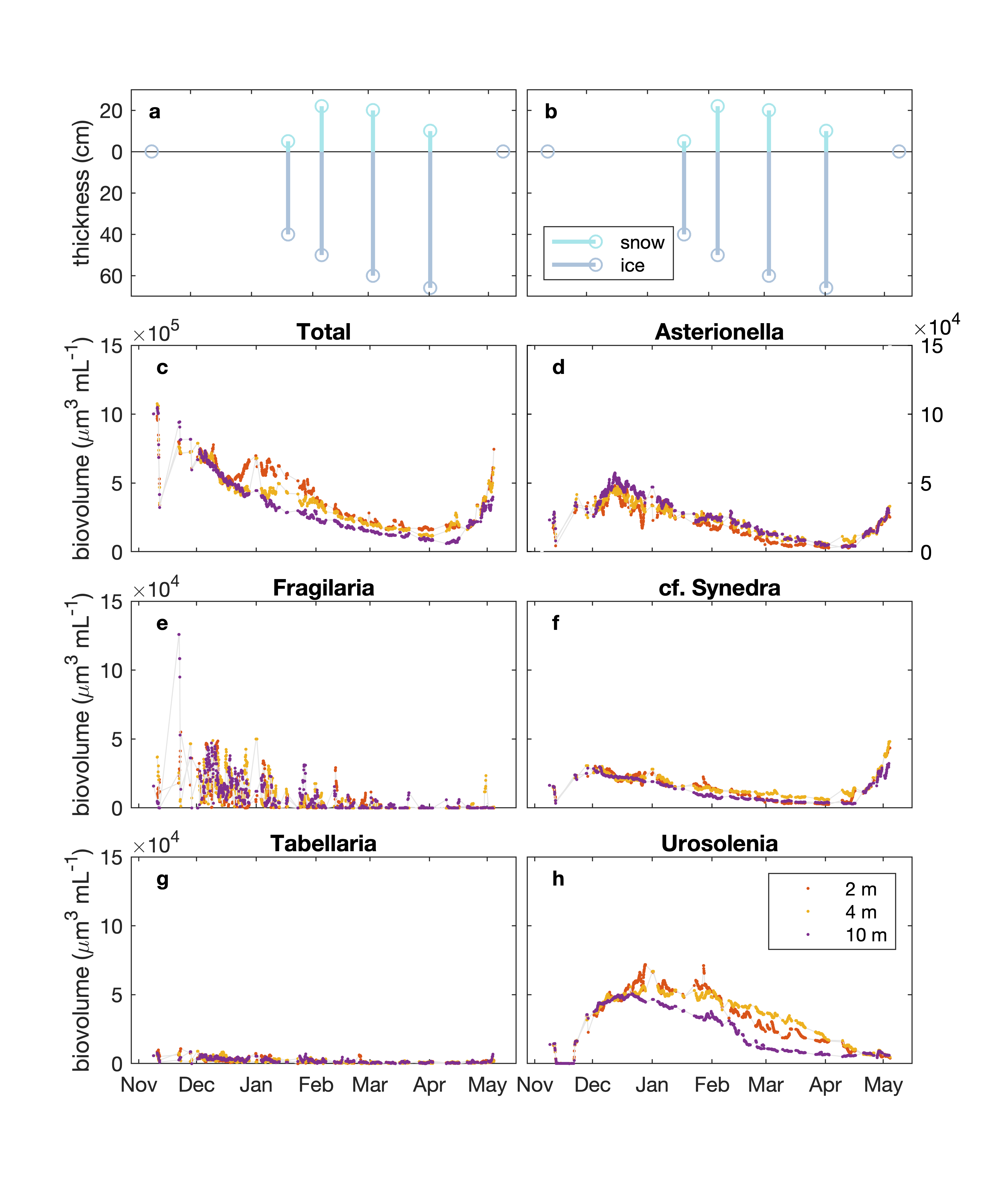


**S4.Figure 1** IFCB-generated biovolume estimates for chlorophyll a-fluorescing particles imaged during the winter of 2014-2015 in Lac Montjoie, to which a ±12h moving average has been applied. **a,b)** Ice and snow thickness, as measured during the GRIL Lake Sentinels sampling programme. **c)** Total biovolumes per mL for imaged particles in each sample: note the factor of ten difference in the y-axis scale relative to the remaining panels, which represent different diatoms. **d)** *Asterionella* biovolumes per mL **e)** *Fragilaria* biovolumes per mL **f)** cf. *Synedra* biovolumes per mL **g)** *Tabellaria* biovolumes per mL **h)** *Urosolenia* biovolumes per mL. Data for 15 m depth are not presented, as sampling frequency was far lower at this depth (roughly 2 samples for every 11 surface samples).
